# Optimizing plasma-derived small extracellular vesicle purification to enhance downstream applications

**DOI:** 10.1016/j.isci.2026.117286

**Published:** 2026-08-22

**Authors:** Fanxiu Ma, Meng Yuan, Yue Zhao, Xiumei Jin, Chengsi Wu, Xin Fang, Zhaodi Jiang, Shuying Liu, Wen-Quan Zou, Zhen Li, Qi Wu, Jiyan Ma

**Affiliations:** 1 Beijing Institute for Brain Research, Chinese Academy of Medical Sciences & Peking Union Medical College, Beijing 102206, China; 2 Chinese Institute for Brain Research, Beijing (CIBR), Beijing 102206, China; 3 National Institute of Biological Sciences, Beijing, China; 4 Department of Neurology, The First Affiliated Hospital, Jiangxi Medical College, Nanchang University, Nanchang, China; 5 Tsinghua Institute of Multidisciplinary Biomedical Research, Tsinghua University, Beijing, China; 6 Department of Neurology, Xuanwu Hospital, Capital Medical University, Beijing, China; 7 Institute of Neurology, Jiangxi Academy of Clinical Medical Science, Department of Neurology, The First Affiliated Hospital, Jiangxi Medical College, Nanchang University, Nanchang, Jiangxi 330006, China

**Keywords:** extracellular vesicles, plasma, ultracentrifugation, density gradient, proteomics

## Abstract

Small extracellular vesicles (sEVs) are functional carriers of biomolecules from host cells, making them highly suitable for early disease diagnosis and monitoring disease progression. However, the purification of sEVs from plasma is complicated by the presence of high levels of proteins, particularly lipoproteins. We have developed an original pretreatment method that effectively removes lipoproteins and fibrinogen, thereby facilitating subsequent sEV purification. Sequential application of ultracentrifugation and optical density gradient on pretreated plasma significantly enhances the purity of sEVs, achieving the removal of over 99% of contaminants and increasing mass spectrometry protein detections by 13.3 and 6.3 times compared to traditional ultracentrifugation and size exclusion chromatography methods, respectively. Furthermore, proteomic analysis verified the validity of sEVs isolated using our approach. Overall, our optimized method substantially improves the purity of plasma sEVs without compromising yield, thereby greatly enhancing their potential for clinical testing and basic biochemical research.

## Highlights

• Pretreatment greatly removes lipoproteins and fibrinogen from plasma
• A 1:20 dilution of the sample improves the removal of lipoproteins by UC
• Combining pretreatment, UC, and ODG (PUD) greatly enhances sEV purity
• The optimized PUD method increases protein detection in mass spectrometry by 13-fold

## Graphical abstract

**Figure:**
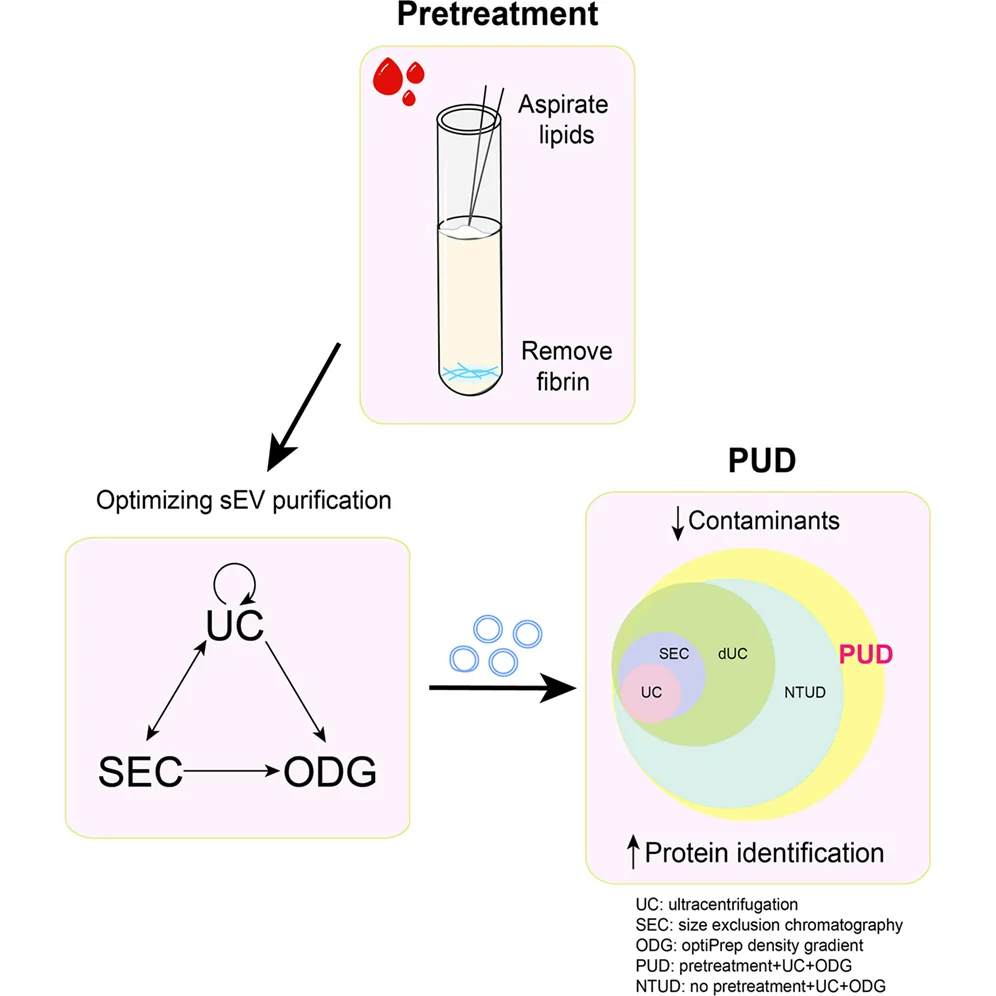

## Teaser

Biochemistry; Proteomics

## Introduction

Extracellular vesicles (EVs) are particles released by cells that are enclosed by a lipid bilayer and cannot replicate.^1^ Small extracellular vesicles (sEVs) specifically refer to EVs that are smaller than 200 nm in diameter and consist of exosomes and small ectosomes, which are also known as microparticles or microvesicles.^1^ Exosomes, which have a diameter of approximately 30–200 nm, are formed in late endosome/multi-vesicular bodies (MVBs) and secreted into the extracellular space through the fusion of MVBs with the plasma membrane.^2^^,^^3^ Ectosomes, which have a diameter of approximately 100–1000 nm, are generated by direct shedding from the plasma membrane.^4^

Peripheral blood is the most used biofluid for liquid biopsy, which contains abundant sEVs that carry protein or RNA molecules derived from host cells. Because the cargo contents of sEVs reflect the metabolic status of host cells, blood sEVs could be a potential source for identifying novel biomarkers, developing effective diagnostic approaches, and potentially uncovering the pathogenic mechanism of diseases.^5^^,^^6^^,^^7^ Indeed, a wide range of blood EV cargo proteins or RNA molecules has already been identified as potential biomarkers for various diseases.^5^^,^^6^ Intriguingly, recent experimental evidence suggests that sEVs derived from the brain can cross the blood-brain barrier. Therefore, tests based on blood sEVs could also be developed for early diagnosis and real-time monitoring of central nervous system disorders,^8^^,^^9^ which are currently diagnosed primarily based on clinical symptoms, physical tests, and neuroimaging.^10^

According to the biophysical characteristics (size, hydrophobicity, density, and immunoaffinity) of sEVs, a variety of sEV isolation methodologies were developed, including size-based techniques: ultracentrifugation (UC), double ultracentrifugation (dUC), size-exclusion chromatography (SEC), and ultrafiltration (UF); hydrophobicity-based technique: precipitation; density-based technique: OptiPrep™ density gradient (ODG); and immunoaffinity-based technique: immunoprecipitation (IP), which are summarized in these review articles.^11^^,^^12^ However, isolation of sEVs from peripheral blood faces several challenges, including the aggregation of EVs and proteins following high-speed centrifugation,^13^^,^^14^ significant contamination with soluble blood proteins like albumin (ALB),^15^^,^^16^ proteins attached to the surface of EVs (corona proteins),^17^ and contamination with lipoproteins.^18^ Lipoproteins pose a particular problem for blood sEVs due to their abundance in circulation and their similarities in size and density to sEVs. Lipoproteins encompass chylomicrons, very low-density lipoproteins (VLDLs), intermediate density lipoproteins (IDLs), LDLs, and high-density lipoproteins (HDLs).^19^ The sizes of VLDL and small chylomicrons and the density of HDL closely resemble those of sEVs.^20^ This similarity presents a significant challenge when using size- or density-based methods to isolate sEVs from blood. Generally, a combination of isolation approaches is necessary to enhance the purity of sEVs,^21^ which is critical for the downstream functional and/or analytical studies of sEVs. For instance, depleting plasma proteins significantly improves the protein coverage in proteomic analysis of blood sEVs,^22^ which is vital for biomarker identification. In RNA-based assays, it has been demonstrated that residual contaminated blood proteins and/or lipoproteins undermine accuracy by binding to RNAs.^23^^,^^24^ Furthermore, lipoproteins can interact with α-synuclein (α-syn) and inhibit the formation of α-syn amyloid fibrils, significantly affecting the widely used real-time quaking-induced conversion (RT-QuIC) assay to detect pathologic α-syn in α-synucleinopathies.^25^ These examples emphasize the need to improve the purity of blood sEVs for their subsequent applications.

In previous plasma/serum sEV studies, investigators have compared two or three methods to identify more effective purification techniques.^26^^,^^27^^,^^28^ However, there is still a lack of systematic comparisons of commonly used purification methods. In addition, further optimization of the existing methodologies is probably required to achieve high purity of sEV isolation from plasma. In this study, we conducted a systematic investigation of plasma sEV purification methods and developed optimized procedures to enhance the purity of sEVs isolated from plasma. We found that pretreating plasma samples with thrombin and implementing a lipid removal step significantly reduce the co-isolated fibrinogen and lipoproteins. For the commonly used sEV isolation methods, our results revealed that precipitation-based techniques or UF are not suitable for isolating plasma sEVs, while dUC, SEC, and ODG are better choices. Additionally, we compared the efficacy of various combinations of these three methods and found that combining a single UC and ODG produces the best purity, removing more than 99% of plasma proteins and lipoproteins, and at the same time, recovers more than 10^9^ EVs. This enhanced purity significantly improves the protein coverage in proteomic analysis with mass spectrometry.

## Results

### Pretreatment of whole plasma samples increases the purity of isolated sEVs

**Figure 1:**
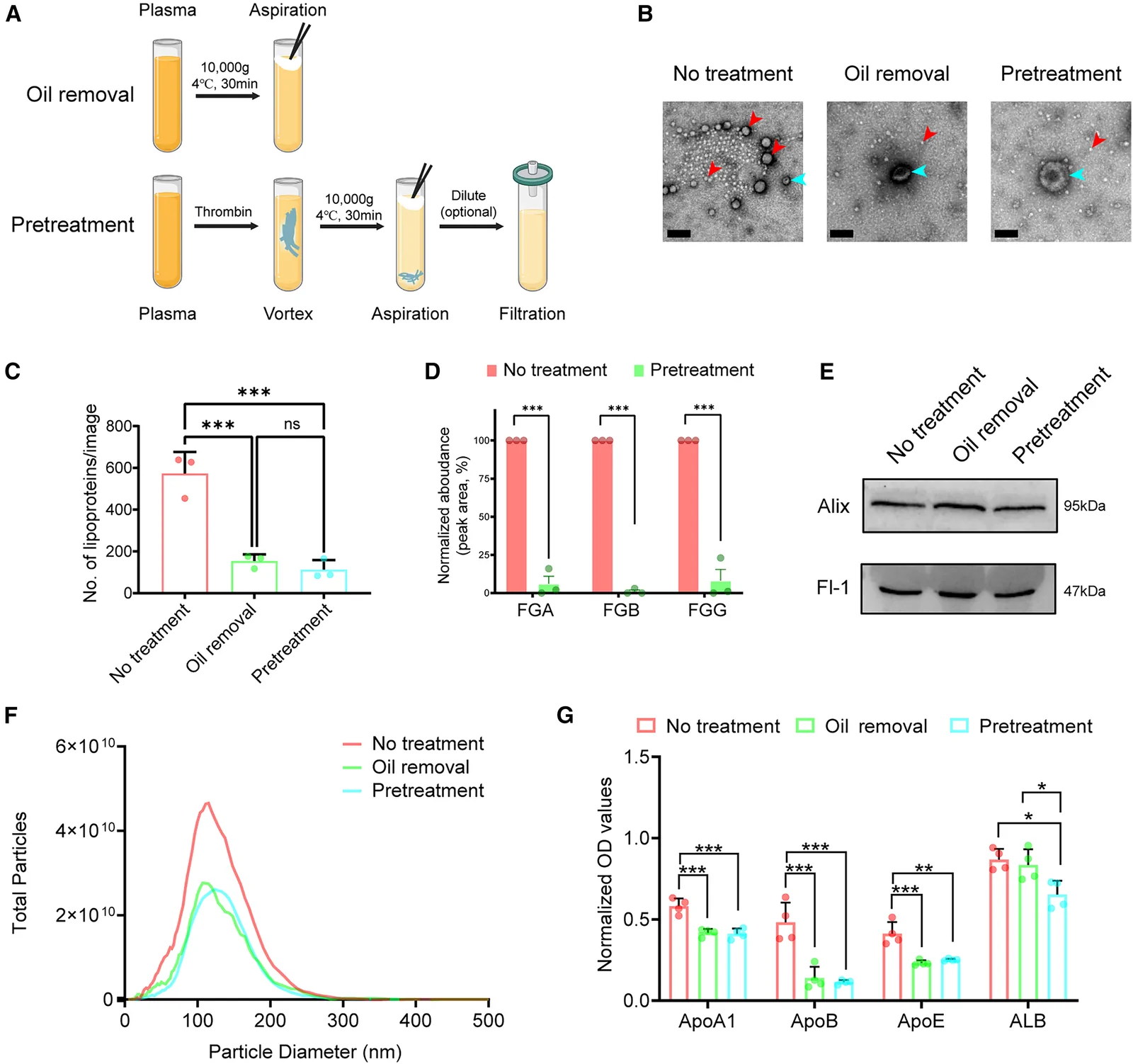
Pretreatment of plasma (A) The schematic of the oil removal and pretreatment procedures. Plasma was subjected to 4 U/500 μL thrombin and vortexed at room temperature for 10 min. Then plasma was centrifuged at 10,000*g* for 30 min at 4°C to pellet fibrin, and the lipids accumulated on top were carefully removed by an aspirator. If UC was employed for sEVs isolation, plasma was diluted by PBS and a 0.45 μm pore size filter was utilized to remove larger particles and residual fibrin from plasma or diluted plasma. sEVs were isolated from non-treated, oil removed, and pretreated plasma. (B) Representative TEM images of sEVs isolated by ultracentrifugation from non-treated plasma (plasma only subjected to a 30 min 10,000 g centrifugation), oil removed plasma (plasma only subjected to a 30 min 10,000*g* centrifugation and removal of lipids), or pretreated plasma (plasma subjected to the full pretreatment). Lipoproteins were indicated by red arrow heads; cyan arrow heads indicated a sEV. (C) Histogram showed the number of lipoproteins in TEM images. (D–F) (D) Three pairs of sEV samples were isolated from non-treated (NT) or pretreated (PT) plasma. Histogram showed the changes in normalized abundance of fibrinogen α chain (FGA), fibrinogen β chain (FGB), and fibrinogen γ chain (FGG) level in mass spectrometry (DDA-MS) analyses. The abundance of each protein in the NT group within each pair was set to 100%. (E and F) EVs were characterized by western blotting with anti-ALIX and anti-fotillin-1 (Fl-1) antibodies (E) and NTA (F). Average size of particles: no treatment – 111.8 ± 45.7 nm, oil removal – 111.1 ± 43.4 nm, pretreatment – 120.00 ± 47.5 nm. (G) ELISA detection of apolipoprotein A1 (ApoA1), apolipoprotein B (ApoB), apolipoprotein E (ApoE), and serum albumin (ALB) in sEV samples. Statistical significance was assessed using one-way ANOVA. Data are represented as mean ± SEM, ∗*p* ≤ 0.05, ∗∗*p* ≤ 0.01, and ∗∗∗*p* ≤ 0.001. Scale bars: 200 nm.

Fibrinogen is a corona protein of plasma EVs that can be converted to insoluble fibrin during centrifugation, contaminating plasma EVs with fibrin aggregates.^17^ To enhance the purity of plasma sEVs isolated from plasma, we implemented a pretreatment procedure as described in the methods section, which includes (1) adding thrombin to convert fibrinogen to fibrin; (2) centrifugation at 10,000*g* to pellet fibrin; (3) removing lipids accumulated on top of the plasma after centrifugation by aspiration (oil removal); (4) dilution and filtration (Figure 1A). After precipitating sEVs by UC, transmission electron microscopy (TEM) analysis showed that the pretreatment step, either with both thrombin treatment and oil removal (pretreatment) or only centrifugation and oil removal, resulted in a significant reduction in lipoproteins, which were observed as solid spherical particles (Figures 1B and 1C). Thrombin treatment removed a substantial amount of fibrinogen (Figure 1D; Table S1). Importantly, the pretreatment did not affect the levels of sEV markers (Figure 1E). Although more particles were detected by nanoparticle tracking analysis (NTA) in sEVs isolated from plasma without pretreatment (non-treated, NT) (Figure 1F), it is important to note that NTA cannot distinguish between EVs and lipoproteins.^29^ Therefore, the decrease in particle count may simply reflect the effective removal of lipoproteins. Consistent with this interpretation, the levels of ApoA1, ApoB, and ApoE were significantly reduced in EVs purified from pretreated plasma (Figure 1G), indicating that the pretreatment procedure effectively reduces lipoproteins.

In conclusion, our pretreatment protocol has substantially enhanced the purity of sEVs. Therefore, we used pretreated plasma for the traditional sEV purification techniques to determine the optimal method for isolating plasma sEVs.

### Precipitation based methods are not well suited for plasma sEVs isolation

A widely used approach for isolating plasma sEVs is based on precipitation, which includes PEG+UC^30^ and a commercially available precipitation reagent called ExoQuick™. We evaluated these two methods and found that PEG+UC sEVs (Figure S1A, indicated by blue arrows) were co-isolated with a large number of lipoproteins (Figure S1A, indicated by red arrows), while no sEVs were detected in samples isolated with ExoQuick in TEM analysis (Figure S1B). Although more than 1 × 10^9^ particles were detected by NTA (Figure S1C), it became evident that a huge number of lipoproteins were co-isolated (Figures S1A, S1B, and S1D), indicating that, in this analysis, the NTA result primarily reflects the presence of contaminated lipoprotein particles rather than sEVs. Furthermore, the total protein content in the EV fraction isolated by ExoQuick was only approximately 25% lower than that of plasma (Figures S1E and S1F), indicating the presence of a substantial amount of plasma proteins. Overall, our results suggest that precipitation-based methods are not suitable for isolating plasma sEVs.

### Optimization of UC and dUC

**Figure 2:**
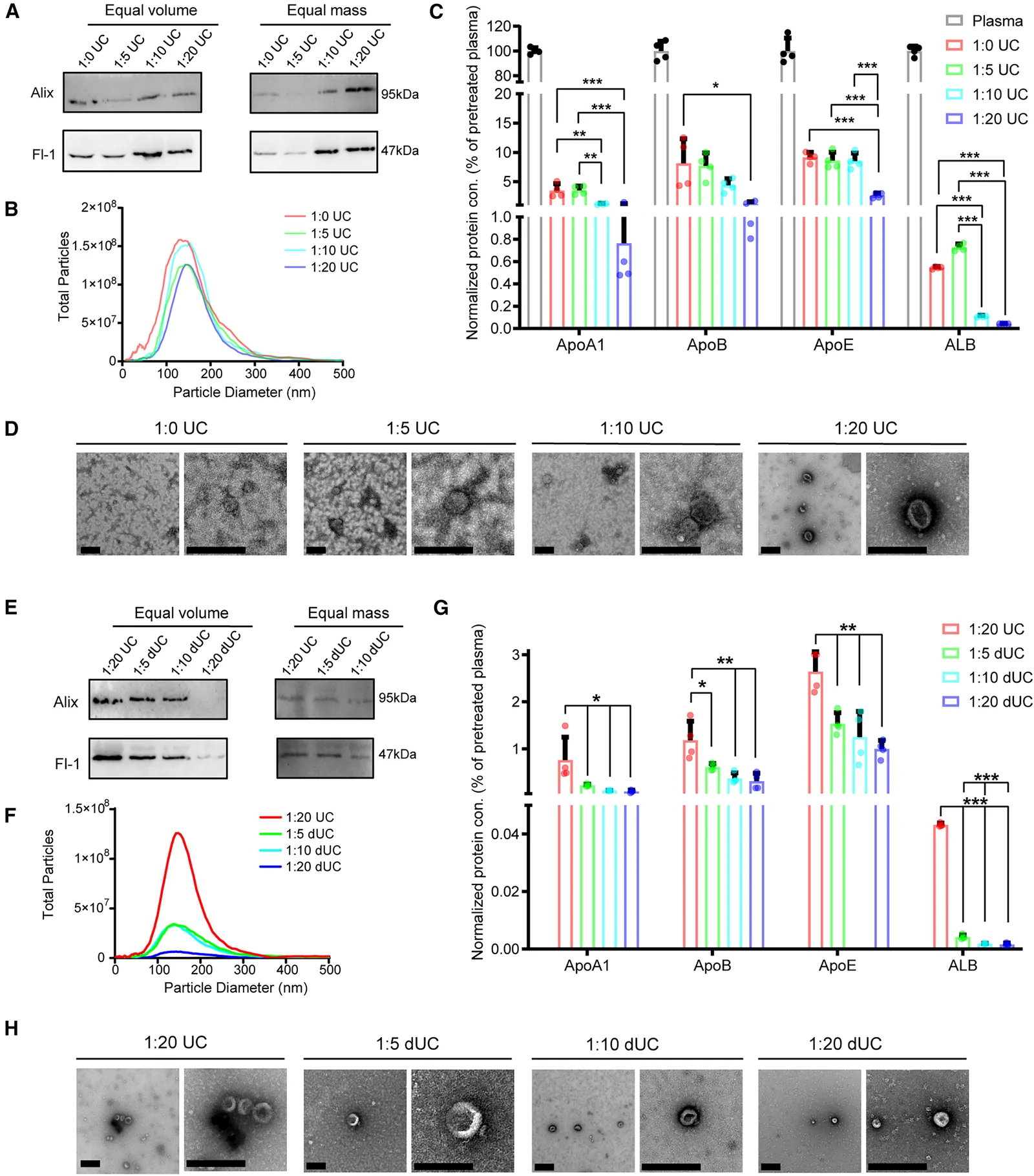
Isolation of sEVs by ultracentrifugation and double ultracentrifugation (A–D) Pretreated pig plasma was diluted with PBS at 1:0 (no dilution), 1:5, 1:10, or 1:20 ratios. sEVs were isolated from diluted/original plasma by ultracentrifugation and characterized by western blotting with anti-ALIX and anti-fotillin-1 (Fl-1) antibodies (A), NTA (B) and TEM (D). Average size of particles: 1:0 UC – 128.3 ± 62.8 nm, 1:5 UC – 146.9 ± 58 nm, 1:10 UC – 150.9 ± 59.8 nm, and 1:20 UC – 149.6 ± 58.2 nm. (C) Histogram indicated the percentage of ApoA1, ApoB, ApoE, and ALB remained after indicated treatments. (E–H) 1:20 UC pellets were resuspended in 2.5 mL (1:5), 5 mL (1:10) or 10 mL (1:20) PBS and then recollected by ultracentrifugation. sEVs were characterized by immunoblotting of indicated sEV protein markers (E), NTA (F) and TEM (H). Average size of particles: 1:5 dUC – 155.2 ± 64.8 nm, 1:10 dUC – 149 ± 61.9 nm, and 1:20 dUC – 157.3 ± 62.4 nm.(G) Histogram indicated the percentage of ApoA1, ApoB, ApoE, and ALB remained after indicated treatments. Statistical significance was assessed using one-way ANOVA. Data are represented as mean ± SEM, ∗*p* ≤ 0.05, ∗∗*p* ≤ 0.01, and ∗∗∗*p* ≤ 0.001. Scale bars: 200 nm.

UC is the most widely used method for isolating sEVs from plasma/serum.^31^ In our initial attempts to isolate sEVs from pretreated plasma using different dilutions, we observed that when an equal volume of EV samples was loaded onto the gel, sEVs isolated with a 1:20 dilution (1:20 UC) exhibited a similar abundance of sEV markers (ALIX and flotillin-1) compared to sEVs isolated without dilution (1:0 UC), suggesting that the diluted samples contained a higher concentration of sEVs. Indeed, when an equal amount of protein was loaded into each well, 1:20 UC EVs exhibited a greater abundance of sEV markers than 1:0 UC EVs (Figure 2A). Moreover, compared to 1:0 UC EVs, 1:20 UC EVs had a comparable number of particles (Figure 2B) and approximately 75% less protein content (Figures S2A and S2B). Consequently, the particle/protein ratio of 1:20 UC EVs was approximately 1-fold higher than that of 1:0 UC EVs (Figure S2C). Additionally, 1:20 UC EVs had a lower number of particles smaller than 30 nm compared to 1:0 UC EVs (Figure S2D) and displayed a significant reduction in lipoproteins and ALB compared to 1:0 UC EVs (Figure 2C). Furthermore, 1:20 UC EVs exhibited clearest lipid bilayer structure in the TEM image (Figure 2D). Collectively, our results recommend a 1:20 dilution before UC for isolating sEVs from plasma.

Next, we examined whether dUC could improve the purity of EVs. The pellet obtained from a 1:20 UC was resuspended in PBS using the same gradient of volumes as the initial UC procedure. Remarkably, dUC effectively removed 80%–95% of proteins and lipoproteins (Figures S2E, S2F, and S2G); however, this process resulted in a loss of 66%–95% of EVs (Figures 2E and 2F), with the highest recovery observed with 1:5 and 1:10 dUC. The particle/protein ratio of 1:10 dUC EVs increased 2-fold compared to 1:20 UC EVs (Figure S2G) and more than 85% of 1:10 dUC EVs had a diameter of less than 200 nm (Figure S2H). Importantly, at all dilutions, dUC EVs exhibited distinct morphological features of EVs (Figure 2H). Therefore, we concluded that a 1:10 dUC can be used for sEVs isolation when purity is more important than the yield of sEVs.

### UF efficiently concentrates sEVs but is not suitable for directly isolating sEVs from plasma

UF is an alternative size-based EVs isolation technique, which offers rapid purification at low centrifugation speeds.^11^ We evaluated the effectiveness of UF for isolating sEVs from plasma. Undiluted and 1:10 diluted pretreated plasma was used to evaluate the possibility of filtration membrane blockage caused by plasma proteins. When these samples were applied to either a 100 kDa Amicon Ultra-0.5 Centrifugal Filter (100K) or 300 kDa Ω Membranes (300K),^32^ we observed that only a few proteins passed through the membrane and appeared in the collecting tube (Figure S3A). Particularly, the undiluted plasma sample caused membrane clogging, resulting in only a 20% reduction in volume after centrifugation. We repeated the centrifugation step multiple times to reduce the volume and observed a sharp drop of volume after the 4^th^ spin in the undiluted plasma sample using the 300K unit, indicating damage to the filtration membrane (Figure S3A). Furthermore, we were unable to detect any sEVs in TEM analysis (Figure S3B). Based on these findings, it is evident that the UF is not suitable for directly isolating sEVs from plasma.

Considering that UF is commonly used to concentrate SEC samples,^33^ we examined its potential for enriching purified EVs. We resuspended the 1:20 UC pellet in 500 μL PBS and concentrated it using 3 kDa (3K) or 100K filters. We found that EV markers, ALIX and flotillin-1, were only detected in the concentrated top fractions (Figure S3C), and the majority of proteins remained in the top fractions as well (Figures S3D and S3E). This confirms that UF can be used to concentrate purified EVs.

### The SEC and UC combination improves the purity of sEVs

**Figure 3:**
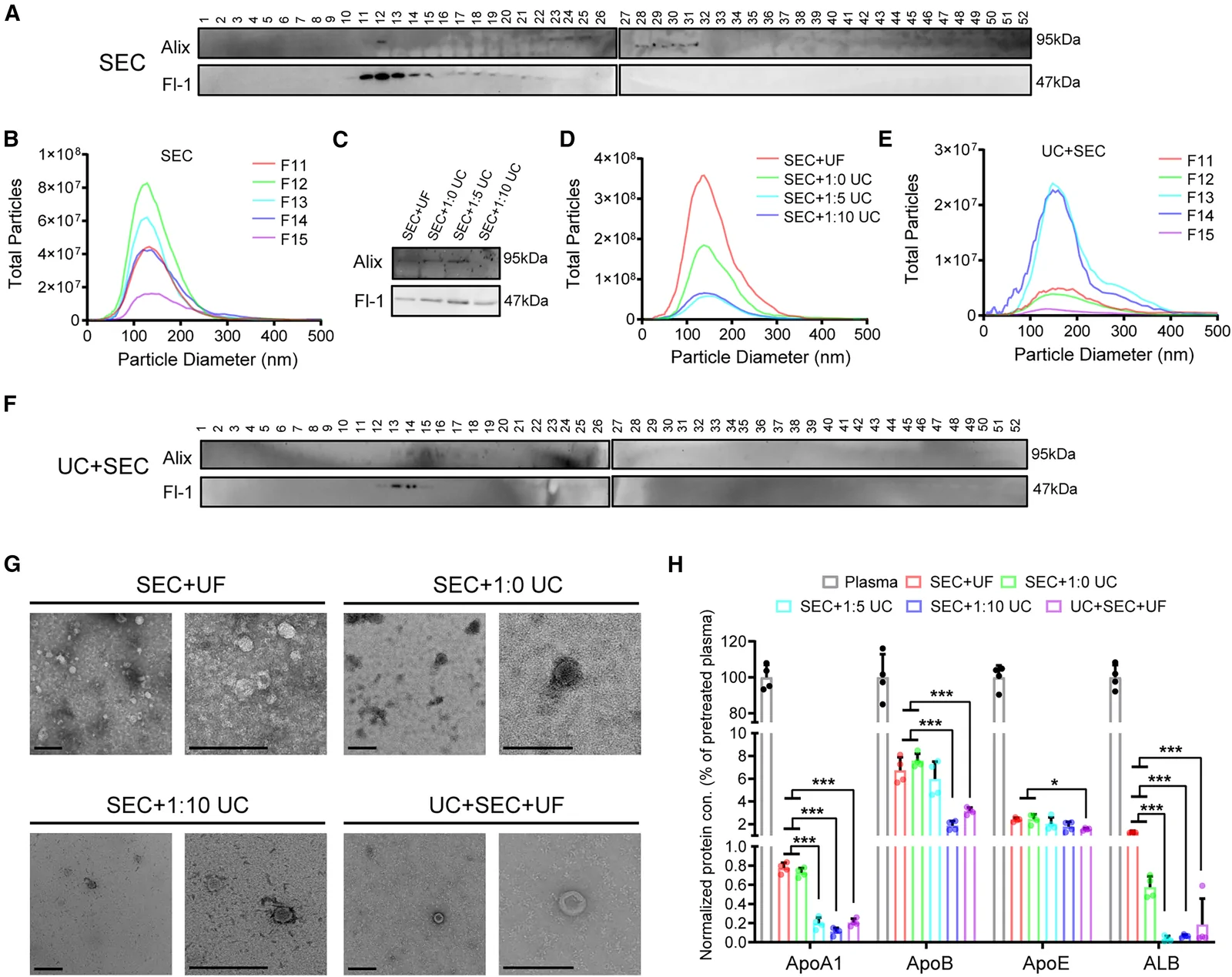
Isolation of sEVs by size-exclusion chromatography and the combination of SEC with UC or ultrafiltration (A–D) sEVs were isolated from pretreated pig plasma by SEC and all fractions were concentrated by 3K centrifugal filter unit (A and B), SEC 11–15 fractions were combined and concentrated by UC or UF as indicated (B–D). sEVs were characterized by immunoblotting of sEV protein markers (A and C) and NTA (B and D). (E and F) 1:20 UC sEVs were loaded to SEC column and all fractions were concentrated by UF. sEVs were characterized by NTA (E) and immunoblotting of indicated sEV protein markers (F). Average size of particles: SEC F11 – 133.1 ± 51.3 nm, SEC F12 – 129.5 ± 46 nm, SEC F13 – 129.4 ± 42.2 nm, SEC F14 – 140.7 ± 64.1 nm, SEC F15 – 149.7 ± 55.3 nm, SEC+UF – 115.8 ± 48.5 nm, SEC+1:0 UC – 148.9 ± 53.1 nm, SEC+1:5 UC – 152.9 ± 52.6 nm, SEC+1:10 UC – 140.3 ± 54.5 nm, UC+SEC F11 – 171.8 ± 70.3 nm, F12 – 142.9 ± 70.4 nm, F13 – 173.1 ± 88.1 nm, F14 – 160.4 ± 67.4 nm, and F15 – 163.3 ± 79.8 nm. (G) The morphologies of sEVs isolated by SEC+UF, SEC+UC or UC+SEC+UF were detected by TEM. (H) Histogram indicated the percentage of ApoA1, ApoB, ApoE, and ALB remained after indicated treatments. Statistical significance was assessed using one-way ANOVA. Data are represented as mean ± SEM, ∗*p* ≤ 0.05 and ∗∗∗*p* ≤ 0.001. Scale bars: 200 nm.

SEC has been reported to isolate EVs directly from plasma with relatively good purity.^33^ We tested this method with our pretreated plasma by loading 500 μL of plasma onto the column and collecting 52 fractions. EV marker ALIX was detected in fractions 11, 12, and 23–31, with a clear peak in fraction 12. Follotin-1 was detected in fractions 10–22, with a peak observed around fractions 11–13 (Figure 3A). Consistent with previous findings, the majority of proteins were eluted in the later fractions (after fraction 19), while only a few proteins were co-eluted with EVs in fractions 11–15 (Figures S4A and S4B). NTA revealed that fraction 12 contained the highest number of particles (Figure 3B), and more than 93% of the particles were in the range of 30–200 nm in diameter (Figure S4C). When the particle/protein ratio was calculated, fraction 12 exhibited the highest ratio, and a significant drop in the ratio was observed in fraction 15 (Figure S4D), suggesting that fractions after 15 contained very few sEVs. Therefore, we pooled fractions 11–15 and utilized it as SEC sEVs.

Since the combined volume of fractions 11–15 was approximately 2.5 mL, a concentrating step with either UF or UC was necessary. We compared two methods and observed that UF retained more EVs than UC (Figures 3C and 3D). On the other hand, UC removed many more proteins and consequently significantly increased the particle/protein ratio of EVs (Figures S4E, S4F, and S4H). The sizes of particles isolated with SEC+UF or SEC+UC were similar, with more than 87% of particles in the range of 30–200 nm in diameter (Figure S4G).

To investigate whether reversing the order of SEC and UC could enhance purification, we applied 1:20 UC samples to SEC. Fractions 13 and 14 exhibited the highest particle counts and highest particle/protein ratios (Figure 3E and S4L), and the sEV marker ALIX and flotillin-1 were detected in fractions 13–18 and fractions 12–15, respectively (Figure 3F). The majority of proteins were eluted in fractions 20–52 (Figures S4I and S4J). In fractions 11–14, only 70–80% of the particles were in the range of 30–200 nm in diameter (Figure S4K). In comparison to SEC EVs (fractions 11–15), UC+SEC EVs contained significantly fewer particles (Figures 3B and 3E), resulting in orders of magnitude lower particle/protein ratio (Figures S4D and S4L).

Lipoprotein analysis revealed that SEC sEVs contained a similar amount of ApoA1, but more ApoB, ApoE, and ALB compared to 1:20 UC EVs (Figures 2C and 3H). The UC+SEC combination effectively removed more lipoproteins and ALB (Figures 3G and 3H), but it also resulted in a significant loss of sEVs (Figure 3E). Therefore, we concluded that UC+SEC is not the optimal choice for purifying sEVs from plasma.

### The combination of UC and ODG is well-suited for plasma sEV isolation

**Figure 4:**
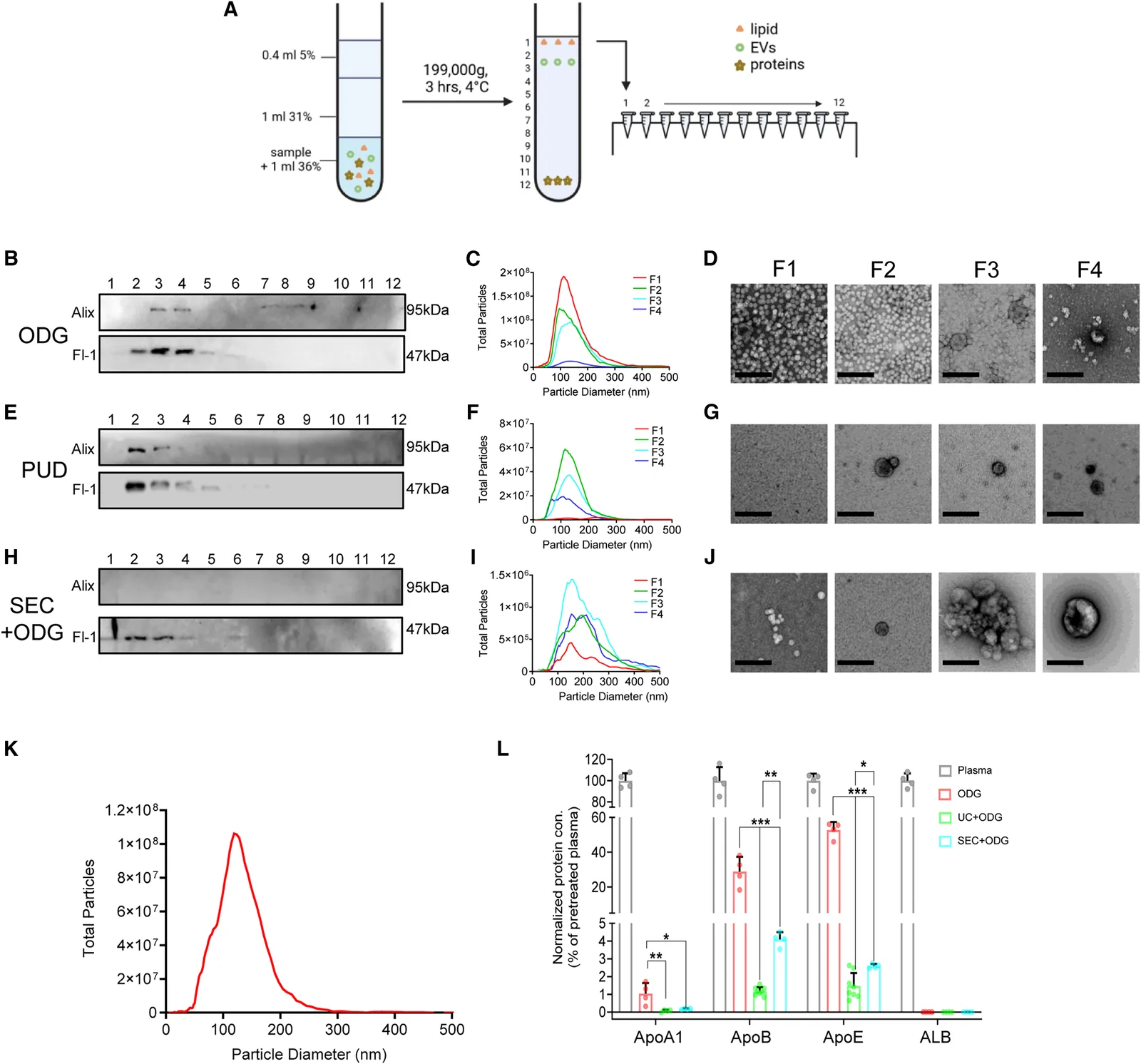
Isolation of sEVs by OptiPrep™ density gradient and the combination of UC or SEC with ODG (A–J) (A)The schematic of the ODG procedures in this study. sEVs were isolated by ODG from pretreated pig plasma (B–D), 1:20 UC sEVs (E–G), or concentrated SEC 11–15 fractions (H–J). sEVs were characterized by immunoblotting of indicated sEV protein markers (B, E, and H), NTA (C, F, and I), and TEM (D, G, and J). Scale bars: 200 nm. Average size of particles: ODG F1 – 127.5 ± 64 nm, ODG F2 – 127.2 ± 60.7 nm, ODG F3 – 140.4 ± 54.1 nm, ODG F4 – 140.1 ± 54.1 nm, PUD F1 – 182.3 ± 94.1 nm, PUD F2 – 133.3 ± 56.2 nm, PUD F3 – 135 ± 77.9 nm, PUD F4 – 137 ± 81.8 nm, SEC+ODG F1 – 171.9 ± 75.4 nm, SEC+ODG F2 – 172.7 ± 71.8 nm, SEC+ODG F3 – 176.1 ± 81.4 nm, and SEC+ODG F4 – 189.7 ± 82.2 nm. (K) 118.6 ± 46.5 nm (B, E, and H) fractions were weight scaled for density evaluation, and the densities (g/mL) of fraction 1 to 12 were (B) 1.046, 1.051, 1.086, 1.102, 1.133, 1.146, 1.158, 1.174, 1.189, 1.208, 1.233, 1.250; (E) 1.042, 1.062, 1.110, 1.134, 1.166, 1.186, 1.202, 1.210, 1.220, 1.240, 1.246, 1.250; (H) 1.022, 1.066, 1.092, 1.109, 1.145, 1.160, 1.171, 1.197, 1.205, 1.216, 1.230, 1.249. UC+ODG fractions 1 to 4 were combined and buffer exchanged to PBS using 3K centrifugal filter unit. The total particles of fractions 1–4 UC+ODG sEVs were measured by NTA. (L) Histogram indicated the percentage of ApoA1, ApoB, ApoE, and ALB remained after indicated treatments. Statistical significance was assessed using one-way ANOVA. Data are represented as mean ± SEM, ∗*p* ≤ 0.05, ∗∗*p* ≤ 0.01, and ∗∗∗*p* ≤ 0.001.

Density gradient is a technique used to separate particles based on their density, allowing for the separation of EVs from VLDLs and LDLs. The traditional ODG protocol requires 16 h of UC,^34^ which is time consuming. We evaluated a shorter ODG protocol originally designed for separating membranes from cytosolic proteins^35^ (Figure 4A). In this protocol, samples are loaded at the bottom of the gradient, and after centrifugation, membrane-containing particles migrate to the top, while other proteins remain at the bottom.

First, we determined the effectiveness of ODG in isolating sEVs directly from plasma. Following centrifugation, EV markers ALIX and flotillin-1 were clearly detected in ODG fractions 3 and 4 (Figure 4B), while the majority of proteins were found in fractions 7–12 (Figures S5A and S5D).

Interestingly, NTA revealed that fraction 1 contained a significantly higher number of particles compared to fractions 2–4 (Figure 4C and S5G), and most of the particles in fractions 1–4 were in the range of 30–200 nm in diameter (Figure S5J). TEM imaging demonstrated that fractions 1–3 contained a large number of lipoproteins while EVs were detected in fractions 3 and 4 (Figure 4D). ELISA analysis further revealed abundant ApoB and ApoE in fractions 1–4 (Figures 4K and S5M). Overall, these findings indicated that fractions 1 and 2 primarily contained lipoproteins, not EVs, while fractions 3 and 4 contained EVs with very low levels of blood proteins but high levels of lipoproteins.

Next, we investigated whether combining UC or SEC with ODG could enhance the purity of sEVs. For the UC+ODG combination, ALIX and flotillin-1 peaked in fractions 2 and 3 (Figure 4E), which also contained the highest number of particles (Figure 4F), predominantly ranging in size from 30–200 nm in diameter (Figure S5K). These fractions displayed higher particle/protein ratios compared to the other fractions (Figure S5H). Regarding the SEC+ODG combination, flotillin-1 was detected in fractions 1–4 and peaked in fractions 2 and 3, but ALIX was not detected (Figure 4H). Fraction 3 had the highest number of particles (Figure 4I) and the highest particle/protein ratio (Figure S5I). The percentage of particles in the 30–200 nm range was similar among fractions 1–4 and lower compared to other preparations (Figures S5J, S5K, and S5L). In both UC+ODG and SEC+ODG combinations, protein levels in fractions 1–4 were significantly reduced compared to UC or SEC (Figures S5B, S5C, S5E, and S5F), while EVs could be clearly detected in fractions 2–4 (Figures 4G and 4J). Importantly, the recovery rate of UC+ODG was order of magnitude higher than that of SEC+ODG (Figures 4F and 4G) and contained significantly fewer lipoproteins (Figures 4G, 4I, S5N, and S5O). We combined fractions 1–4 of UC+ODG sEVs, performed buffer exchange to PBS using a 3K centrifugal filter unit and confirmed the presence of EVs by NTA (Figure 4K). ELISA analysis revealed that UC+ODG removed more than 99% of ApoA1, ALB, and more than 98% of ApoB and ApoE, a significant improvement compared to ODG alone or the SEC+ODG combination (Figure 4L).

Our results support that the UC+ODG approach is well-suited for isolating sEVs from pretreated plasma.

### Pretreatment enhances the proteomic detection of cargo proteins of plasma sEVs

**Figure 5:**
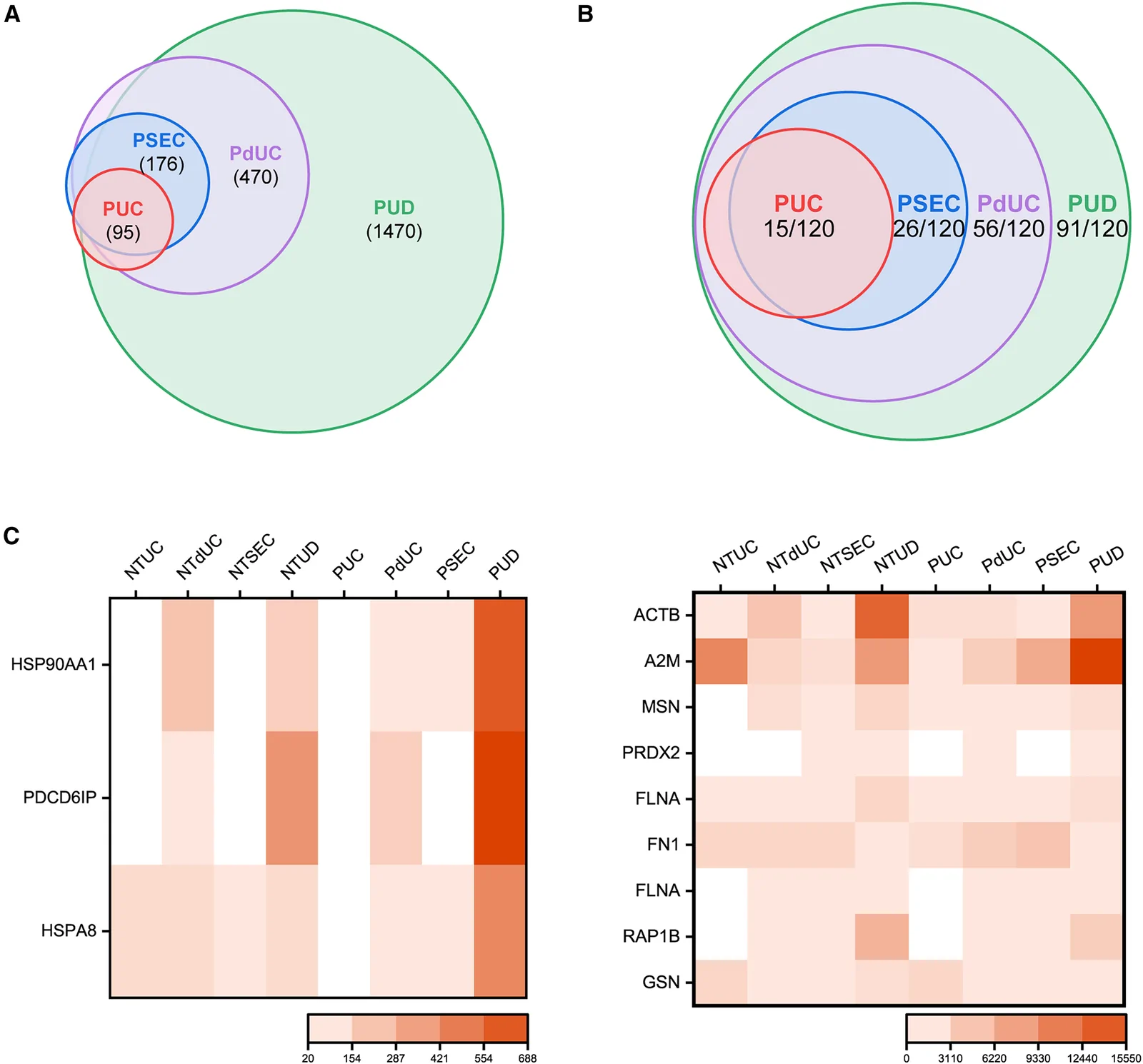
Proteomic analyses of isolated sEVs (A and B) EV proteins were detected by mass spectrometry (DIA-MS).(A) The number of proteins identified and the number of commonly detected sEV proteins (B) in sEVs isolated from pretreated plasma with 1:10 UC, 1:10 dUC, SEC, or UC+ODG. Notably, 91 out of the 120 commonly detected proteins were identified in PUD sEVs. (C) The diagrams indicated the abundance of EV main (left) and sub (right) markers that were detected in sEVs from different isolation methods.

To assess whether our improved isolation method can improve proteomic analysis of plasma sEVs, we compared the proteomic profiles of sEVs isolated using our optimized method (pretreated plasma subjected to UC+ODG, denoted by PUD) with those isolated with other techniques, specifically pretreated plasma subjected to UC, dUC, or SEC. All samples were processed at the same time and subjected to the same mass spectrometry. We identified 1,470 proteins using the PUD method, compared to 95, 176, and 470 proteins detected by other methods (Figures 5A; Table S2). The number of proteins detected in sEVs isolated by PUD is 3–15.5 times greater than that of the other methods. Commonly detected sEV proteins were listed in two databases, ExoCarta top 100 sEV proteins and The Minimal Information for Studies of Extracellular Vesicles 2023 (MISEV2023). The combination of these two databases resulted in a total of 120 proteins commonly present in sEVs. Notably, 91 out of the 120 proteins were identified in sEVs isolated by PUD (Figures 5B; Table S3), and among the 100 proteins listed in ExoCarta top 100, 86 proteins were found in sEVs isolated by PUD (Figure S6D; Table S4).^12^^,^^36^ While some sEV markers, particularly the main markers and sub-markers, were absent in sEVs isolated using UC, SEC, or dUC (Figure 5C).^37^

To determine the effectiveness of our original pretreatment in enhancing the recovery of EV proteins, we compared the proteomic profiles of sEVs isolated by PUD with those isolated using various methods without pretreatment. We found that, even in the absence of pretreatment, UC+ODG (NTUD) recovered a greater number of proteins than other methods (Figure S6A). However, the pretreatment significantly increased the total number of proteins identified. For proteins, commonly detected and ExoCarta top 100 sEV proteins, both PUD and NTUD performed better than other techniques (Figures S6B and S6C; Tables S3 and S4), but PUD sEVs contained highest abundance of main EV markers (Figures 5C; Table S5), indicating an improvement in purity.

Together, our findings demonstrate that the PUD combination is capable of isolating sEVs with high purity and significantly improves the sensitivity of proteomic analysis.

### Isolation of human plasma sEVs using PUD

**Figure 6:**
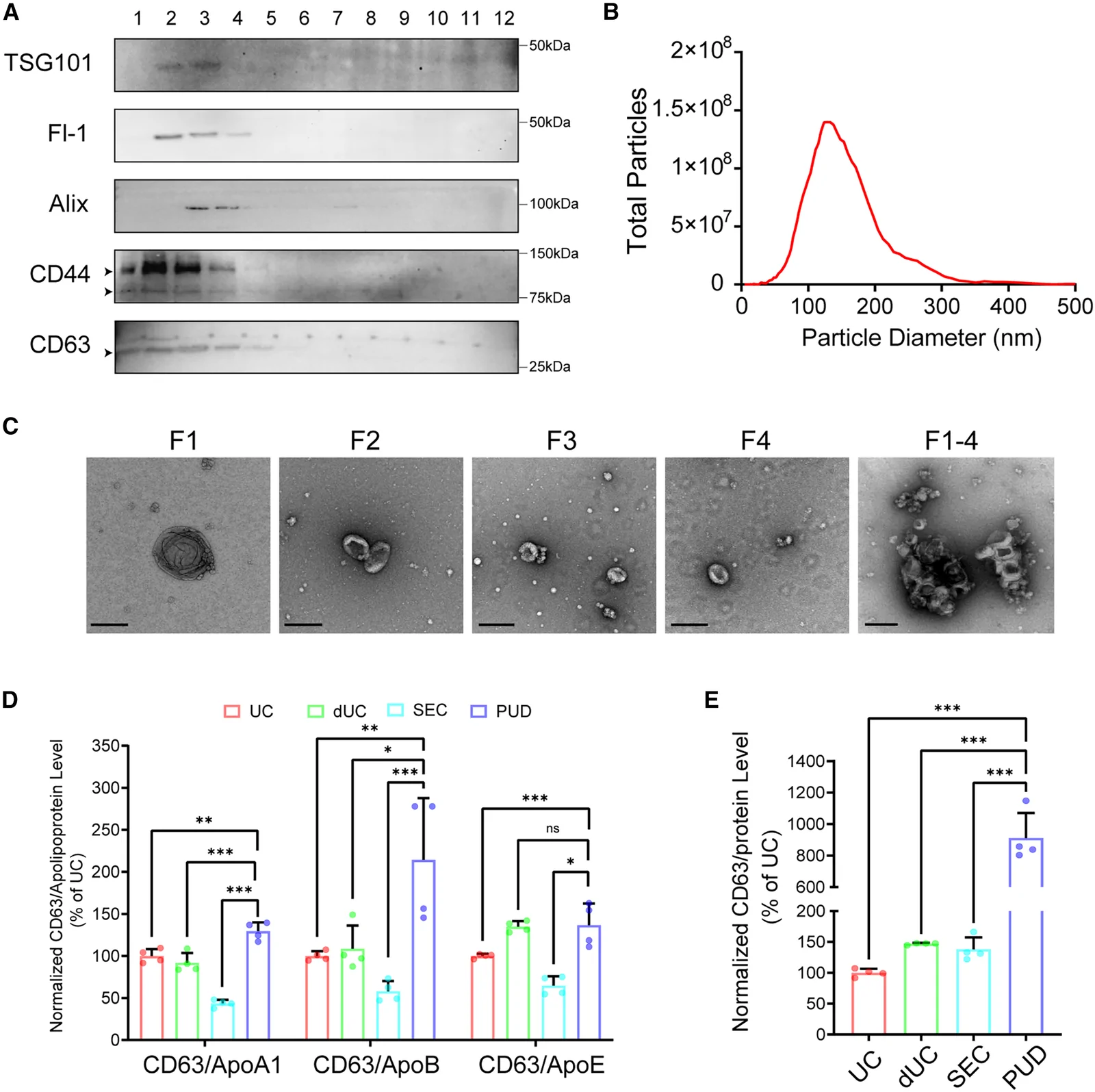
Isolation of human sEVs by PUD (A–C) sEVs were characterized by immunoblotting of indicated sEV protein markers (A), NTA (B), and TEM (C). (B) The average size of particles is 138.14 ± 55.1 nm. Scale bars: 200 nm. (A) Fractions were weight scaled for density evaluation, and densities (g/mL) of fraction 1 to 12 was 1.008, 1.021, 1.060, 1.098, 1.153, 1.172, 1.183, 1.194, 1.203, 1.219, 1.252, and 1.280. (D and E) Histogram indicated the CD63/apolipoprotein ratios (D) and CD63/protein ratios (E) in sEVs isolated from human plasma with 1:10 UC, 1:10 dUC, SEC, or PUD. Statistical significance was assessed using one-way ANOVA. Data are represented as mean ± SEM, ∗*p* ≤ 0.05, ∗∗*p* ≤ 0.01, and ∗∗∗*p* ≤ 0.001.

To determine the applicability of our protocol to human plasma, we utilized our PUD method to isolate sEVs from human plasma. As shown in Figure 6A, EV markers TSG101 and flotillin-1 were detected in fractions 2–4 of the density gradient, which were separated from the majority of proteins (mainly in fractions 5–12) (Figures S7A and S7B). Pooled fractions 1–4 was subjected to TEM and NTA analyses (Figures 6B and 6C), which confirmed the purity and yield of sEVs isolated from human plasma. ELISA results demonstrated that less than 0.2% of ApoA1, ApoB, and ApoE remained after PUD purification (Figure S7C). The ratio of CD63/apolipoproteins and CD63/proteins were used to compare the effectiveness of various methods to enrich sEVs and remove apolipoproteins and plasma proteins. The PUD-isolated sEVs had higher CD63/apolipoprotein and CD63/protein ratios than that of sEVs isolated by other methods, confirming that PUD is a better approach for isolating plasma sEVs (Figures 6D and 6E).

## Discussion

Blood sEVs have great potential for disease diagnosis and biomarker development. However, isolating EVs from blood has always been challenging. Despite various attempts,^32^^,^^33^^,^^38^ a practical procedure that can efficiently isolate blood EVs with high purity is still lacking, hindering their wide applications to many respects including biomarker development. In this study, we systematically investigated methods for isolating sEVs from plasma and demonstrated that our optimized PUD protocol is able to isolate them with high purity and high yield. Specifically, we developed a pretreatment step that can greatly reduce various contaminants such as lipoproteins and fibrinogens without their significant loss (Figure 1). The pretreatment step robustly improves the purity of plasma isolated sEVs. Using the pretreated plasma, we evaluated various sEV isolation techniques and identified the combination of UC and ODG as the most effective in removing contaminated lipoproteins and blood proteins without sacrificing the yield of sEVs. Additionally, our study also revealed that: (1) UC with a 1:10 to 1:20 dilution can remove more proteins and lipoproteins compared to undiluted UC; (2) UF is not suitable for direct plasma sEV isolation, but it can be used to concentrate sEVs after other procedures; (3) SEC can remove a large number of proteins, but a non-negligible amount of lipoproteins still remains with isolated sEVs; (4) the particles/proteins ratio of sEVs obtained from UC+ODG was markedly higher than all other methods, while the levels of lipoprotein markers were relatively lower; and (5) precipitation-based methods are not suitable for sEV isolation from plasma (Table 1).

**Table 1:** Comparison between different isolation methods for sEVs from plasma samples

Method	ALB remained (%)^a^	Lipoprotein remained (%)^b^	Yield^c^	Number of proteins detected by DIA-MS	Number of EV proteins detected by DIA-MS^d^	Number of ExoCarta top 100 sEV proteins detected by DIA-MS	Time required(minutes)
PEG+UG	0.85	–	3.00	–	–	–	140+overnight
ExoQuick	5.68	–	3.00	–	–	–	85.00
1:0 UC	0.55	6.92	3.00	–	–	–	120.00
1:5 UC	0.73	6.60	2.00	–	–	–	120.00
1:10 UC	0.11	4.67	3.00	95.00	15.00	15.00	120.00
1:20 UC	0.04	1.53	3.00	–	–	–	120.00
1:5 dUC	0.00	0.79	2.00	–	–	–	200.00
1:10 dUC	0.00	0.58	2.00	470.00	58.00	57.00	200.00
1:20 dUC	0.00	0.47	1.00	–	–	–	200.00
SEC	1.27	3.30	2.00	176.00	26.00	26.00	80.00
SEC+1:0UC	0.58	3.59	2.00	–	–	–	150.00
SEC+1:5UC	0.04	2.74	2.00	–	–	–	150.00
SEC+1:10UC	0.07	1.26	1.00	–	–	–	150.00
UC+SEC+UF	0.19	1.64	2.00	–	–	–	175.00
ODG	0.00	–	2.00	–	–	–	235.00
SEC+ODG	0.00	2.29	1.00	–	–	–	270.00
UC+ODG	0.00	0.90	3.00	1470.00	91.00	86.00	310.00

a The percentage of pretreated plasma.

b The percentage of average value of ApoA1, ApoB, and ApoE of pretreated plasma.

c Yield is estimated based on western blot, NTA, and TEM results.

d The database of commonly detected EV proteins was compiled from ExoCarta top100 and MISEV2023, after removing duplicated proteins, a total of 120 proteins were listed.

UC is widely used as the gold standard for isolating sEVs.^32^^,^^38^ However, when we attempted to use UC to isolate sEVs from plasma, a high amount of proteins were co-isolated (Figure S2B). In contrast, when UC was used to isolate sEVs from serum-containing cell culture media, the amounts of co-isolated proteins were much lower.^39^ The high abundance of proteins and lipoproteins in the blood likely contributes to the co-precipitating of blood proteins during UC. Therefore, by diluting the plasma before UC, fewer proteins were precipitated, resulting in higher purity. The 1:20 UC method successfully removed more than 99% of apolipoprotein A1 which indicated that this method can efficiently separate HDL from sEVs. Considering that the density of HDL is similar to that of sEVs, this step is likely critical before the density-based ODG.

SEC is a time-efficient method, and we found that it can isolate a higher number of particles and effectively remove a large amount of contaminated blood proteins and lipoproteins (Figure 3). However, it is not our preferred method for isolating sEVs from plasma for the following reasons. Firstly, as previously reported, SEC sEVs contain a higher amount of apolipoprotein B, indicating the co-isolation of more LDL and VLDL.^26^^,^^33^ Since the concentration of EVs in blood is believed to be between 10^7^ and 10^9^ particles/mL, while lipoproteins are around 10^16^ particles/mL,^40^ a significant portion of the particles isolated by SEC may actually be co-isolated lipoproteins. Secondly, consistent with a previous report,^33^ the peak of sEV markers in SEC is quite broad, and we detected them spanning from fraction 11 to fraction 31 (Figure 3A). Due to the sharp decline in the particles/protein ratio, we only collected fractions 11–15 to achieve high purity, but a large portion of sEVs in other fractions was unrecovered. Thirdly, the concentrations of sEVs isolated by SEC are quite dilute, requiring a concentration step for further analysis. Both UF and UC are suitable for concentrating SEC sEVs. We chose UF because it is relatively faster and can recover a higher number of particles. However, SEC+UC, especially UC with dilutions, can effectively remove more contaminated lipoproteins and other proteins in the plasma.

Density gradient centrifugation using sucrose or Optiprep™ is the only density-based technique for isolating sEVs, which typically involves prolonged UC.^28^^,^^34^ Our protocol significantly reduces the centrifugation time to just 3 h and employs a simplified 3-layered Optiprep™ gradient that efficiently separates sEVs from the majority of proteins. For plasma sEV isolation, the 1:20 UC is the most effective for removing lipoproteins, but ineffective in removing some plasma proteins such as ALB. While ODG is the best method for protein removal, it does not separate sEVs from LDL or VLDL. SEC still retains a substantial number of lipoproteins and other proteins (Table 1). Given that plasma samples contain high levels of both lipoproteins and other plasma proteins, combining 1:20 UC for lipoprotein removal followed by ODG for protein removal represents the optimal approach for isolating plasma sEVs. Notably, a similar setting, dUC+ODG combination, was used in a recent study to isolate EVs entrapped in fibrocalcific tissues,^41^ suggesting that our UC+ODG protocol may also be applicable to tissue sEV isolation. However, the entire PUD process takes over 6 h and requires training for the experimental personnel. Therefore, the choice of isolation method depends on the required purity of sEVs for subsequent applications.

Overall, our thorough study of plasma sEV isolation revealed the advantages and disadvantages of each technique and developed an optimized plasma sEV isolation protocol. The validity and applicability of this approach have been confirmed through proteomic analysis of isolated sEVs. Our findings will greatly facilitate the application of plasma sEVs in clinical and basic research.

### Limitations of the study

This study has several limitations. First, sEVs are susceptible to damage or disruption during UC. Therefore, only freshly prepared plasma or plasma stored at −80 °C may be used for the PUD process. Second, to date, our research has focused on isolating high-purity plasma sEVs, and has not yet assessed their value for disease detection. Further studies are needed to validate that PUD-purified sEVs are suitable for disease detection. Finally, because the ultracentrifuge has a limited sample-loading capacity, PUD is not suitable for high-throughput purification of plasma sEVs. Consequently, further improvements are required to support its potential application in clinical applications.

## Resource availability

### Lead contact

Further information and requests for resources and reagents should be directed to and will be fulfilled by the lead contact, Jiyan Ma (majiyan@cibr.ac.cn).

### Materials availability

This study did not generate new unique reagents.

### Data and code availability

• This paper does not report original code.
• The mass spectrometry proteomics data have been deposited to the ProteomeXchange Consortium (https://proteomecentral.proteomexchange.org) via the iProX partner repository. The data are publicly available as of the date of publication. Accession numbers are listed in the key resources table.
• Any additional information required to reanalyze the data reported in this paper is available from the lead contact upon request.

## Acknowledgments

This work was supported by internal funding from the Chinese Institute for Brain Research, Beijing (CIBR). Figure 1A was created in BioRender (M.F., 2026) https://BioRender.com/800jolr. Figure 4A was created in BioRender (M.F., 2026) https://biorender.com/p3pz5a9.

## Author contributions

Data curation, F.M., Q.W., Y.Z., and Z.L.; formal analysis, F.M., Q.W., Y.Z., and J.M.; investigation, F.M., Q.W., and M.Y.; methodology, F.M., Q.W., Y.Z., X.J., Z.J., Z.L., and J.M.; validation, F.M., Q.W., M.Y., and X.J.; visualization, F.M. and Q.W.; writing – review and editing, F.M., Q.W., Y.Z., W.-Q.Z., Z.L., and J.M.; writing – original draft, Q.W.; resources, X.J., X.F., Z.J., S.L., W.Z., and J.M.; supervision, Z.J., S.L., W.-Q.Z., Z.L., and J.M.; project administration, J.M; funding acquisition, J.M.; conceptualization, J.M.

## Declaration of interests

The authors declare no competing interests.

## STAR★Methods

### Key resources table

**Table:** 

REAGENT or RESOURCE	SOURCE	IDENTIFIER
**Deposited data**		

mass spectrometry proteomics data	iProX	PXD075798

**Software and algorithms**		

GraphPad Prism	GraphPad Software Inc.	www.graphpad.com/features
Origin	Origin Lab	www.originlab.com/

**Other**		

Sorvall MX150+ micro-ultracentrifuge	Thermo Scientific	#50135641
S80-AT3 fixed angle rotor	Thermo Scientific	#45590
S55-S Swinging-Bucket Rotor	Thermo Scientific	#45594
OptiPrep	Serumwerk Bernburg	#1893
100K Amicon Ultra-0.5	EMD Millipore	#UFC5100

### Experimental model and study participant details

#### Plasma collection

Erythrocytes are highly susceptible to rapid breakdown after collection of whole blood samples from living organisms. This can lead to contamination of the isolated small extracellular vesicles with cytoplasmic organelles and proteins. To prevent hemolysis, it is crucial to promptly generate plasma or serum from the whole blood samples in order to remove platelets, cell debris, and other blood cells.^31^^,^^42^

To standardize EV isolation protocol, pig plasma (#SBJ-P-P001) was purchased from SBJ Biological Technology. Plasma was obtained by two times centrifugation at 2,500 g, room temperature for 15 min. Plasma was stored at −80°C or directly proceed to EV isolation with a maximum of only one freeze-thaw cycle.

Human venous blood was collected in ethylenediaminetetraacetic acid (EDTA) tubes. Plasma was obtained by centrifugation at 2,500 g, 4 °C for 10 min. Plasma was store at −80°C or directly proceed to EV isolation. A maximum of only one freeze-thaw cycle was performed. Three plasma samples were obtained from: a 45-year-old male, a 32-year-old female and a 26-year-old female. The study was monitored and approved by the Project Management Office of Investigator-initiated Trials of The First Affiliated Hospital of Nanchang University (Ethical Approval No: [2023]145) and the Ethics Committee of Xuanwu Hospital of Capital Medical University (Ethical Approval No: [2020]035). All subjects gave their written informed consents to the study.

### Method details

#### Pretreatment

Four units of thrombin (Solarbio #9002-04-4) were added to 500 μL of either pig plasma. The mixture was immediately vortexed at room temperature for 10 min (min) to convert fibrinogen to fibrin. Subsequently, the plasma was centrifuged at 10,000g for 30 min at 4 °C to pellet fibrin, and the lipids that accumulated on top were carefully removed by an aspirator. The supernatant was then transferred to a new tube. For samples subjected to ultracentrifugation, supernatant was diluted with 5, 10, and 20 volumes of Phosphate-buffered saline buffer (PBS). Then, a 0.45 μm pore size filter was utilized to remove larger particles and residual fibrin from plasma or diluted plasma.

#### Ultracentrifugation

The pretreated plasma was centrifuged in a thick-walled tube (Fisher Scientific #45595) at a speed of 100,000 g for 70 min in an S80-AT3 fixed angle rotor (Thermo Scientific #45590) at 4 °C using a Sorvall MX150+ micro-ultracentrifuge (Thermo Scientific #50135641). For double UC (dUC), the pellets collected from the first centrifugation (UC EVs) were resuspended with 2.5–10 mL of PBS and then centrifuged at 100,000 g for 70 min at 4 °C. the resulting pellets were resuspended in PBS and used as dUC EVs.

#### Precipitation based isolation

EVs were precipitated from pretreated plasma using Polyethylene glycol (PEG) with M_n_ (number average molecular weight) of 6000 (Solarbio #P8250)^30^ or ExoQuick™ (System Biosciences #EXOQ5A-1). Specifically, the pretreated plasma was mixed with 12% PEG6000 in 1M sodium chloride solution at a ratio of 1:1 or ExoQuick reagent at a ratio of 1:4. To prepare PEG+UC EVs, the mixture was incubated overnight at 4 °C. EVs were pelleted at 15,000 g, 4 °C for 30 min. The pellet was resuspended in in 5 mL of PBS and ultra-centrifuged at 100,000 g for 70 min at 4 °C. The ExoQuick mixture was incubated 30 min at 4 °C and centrifuged at 1,500 g, 4 °C for 30 min.

#### Ultrafiltration (UF)

Pretreated plasma, SEC, or ODG collections were concentrated or buffer exchanged using the following centrifugal filter units: 3K Amicon Ultra-0.5 (EMD Millipore #UFC5003), 100K Amicon Ultra-0.5 (EMD Millipore #UFC5100), 300K Omega Membrane (Pall Corporation #OD300C35), or 5K Vivaspin 20 Ultrafiltration unit (Sartorius #VS2011). The centrifugation was performed at 14,000 g (for the Amicon Ultra unit and Omega Membrane) or 6,000 g (for the Vivaspin unit) at 4 °C for 15 min.

#### Size exclusion chromatography (SEC)

The SEC column was filled with Sepharose CL-6B resins (Solarbio #S8751). Specifically, 500 μL of the sample was loaded onto the equilibrated 20 mL SEC column to allow it to fully drip out by gravity flow. PBS was added, and the first 500 μL PBS elution was designated as fraction 1. A total of 52 fractions of 500 μL each were collected.^33^ Fractions 11–15 were mixed and concentrated using 3K Amicon Ultra-0.5 centrifugal filter units.

#### OptiPrep™ density gradient

Pretreated plasma, UC sEVs, or SEC sEVs were mixed with a stock iodixanol solution (OptiPrep, Serumwerk Bernburg #1893) to generate 1 mL of 36% iodixanol solution, and the mixture was loaded at the bottom of a 2.2 PA ultracentrifuge tube (Fisher Scientific #45240). Then, 1 mL of 31% iodixanol solution and 0.4 mL of 5% iodixanol solution were loaded on top sequentially (Figure 4A). The gradient was centrifuged at 199,000g for 3 h (8) at 4 °C using an S55-S Swinging-Bucket Rotor (Thermo Scientific #45594). Twelve 200-μL fractions were collected from top to bottom, and the density of each fraction was assessed by weighing.^35^ Fractions containing sEVs were pooled and concentrated to 100 μL using a 3K Amicon Ultra-0.5 centrifugal filter unit.

#### Western blot analysis and coomassie blue staining

Western blotting was performed as previously described.^43^ In brief, the protein concentration of sEVs from different experimental paradigms was measured by Pierce bicinchoninic acid (BCA) Protein Assay (Thermo Scientific #23227) following the manufacturer’s instructions. An appropriate amount of sEVs was lysed with radioimmunoprecipitation lysis buffer (RIPA buffer, Beyotime #P0013C). Denatured samples were separated on 8% or 10% sodium polyacrylamide gels, transferred to polyvinylidene difluoride membranes, blocked with 5% milk, and incubated overnight at 4°C with anti-Alix (Santa Cruz Biotechnology #sc-53540), flotillin-1 (Cell Signaling Technology #18634), CD63 (Abcam #ab134045), CD44 (Abcam #157107) or TSG101 antibodies (Abcam #ab125011).^44^^,^^45^ The membranes were subsequently incubated with horseradish peroxidase-conjugated secondary antibodies (1:5,000) (Biorad # 1706515, 1706516), and the immunoreactive proteins were detected using an ECL kit (Tannon #180–501). Afterward, membranes were stained with Coomassie blue (Solarbio #8430) to visualize total proteins.

#### Nanoparticle tracking analysis (NTA)

NTA was conducted using a Zetaview Basic PMX-120 (Particle Metrix, Germany), following the instructions provided by the manufacturer. EVs were diluted with PBS and injected using a 5-mL syringe. Videos were recorded and analyzed using the NTA software provided by the manufacturer to determine the size and concentration of particles.

#### Transmission electron microscopy (TEM)

The copper grids (Zhong Jing Ke Yi # BZ110135a) coated with formvar film and a thin layer of carbon film (∼10 nm) were glow-discharged for 1 min. Next, 10 μL of the solution containing purified EVs was deposited on the copper grids. After 1 min, any excess solution was blotted away with a piece of filter paper, and the grid was washed three times with double-distilled water (ddH_2_O). For negative staining, the grids were rinsed with three drops of 10 μL of 2% uranyl acetate (UA) (Electron Microscopy Sciences #22400) and the last drop was left on the grid for 1 min. Any excess solution was blotted away, and the grid was air dried for TEM analysis.

EM images were recorded on a Tecnai G2 Spirit Twin electron microscope (Field Electron and Ion Company, FEI) operating at 120 kV. The images were recorded with a 4k x 4k 895 CCD camera (Gatan Corp).

#### The enzyme-linked immunosorbent assay (ELISA)

The ELISA assay was performed as previously described with minor modifications.^46^ Maxisorp 96-well plates (Thermo Fisher #439454) were coated overnight at 4°C with diluted pretreated plasma or sEVs in sodium bicarbonate buffer pH 8.2. Residual protein binding sites in the well were blocked with 2% skim milk in PBS for 2 h at room temperature. Antibodies against ApoA1 (Cloud-Clone Corp. #PAA519Po02 for pig samples), ApoB (Cloud-Clone Corp. #PAC003Po01 for pig samples), ApoE (Cloud-Clone Corp. #PAA704Po01 for pig samples), or ALB (Cloud-Clone Corp. #PAB028Po01 for pig samples) were diluted and incubated with the samples for 2 h at room temperature. The wells were then washed and incubated with 1:3,000 dilution of goat anti-rabbit HRP (Proteintech #SA00001) for 1.5 h. After washing, 100 μL of ultra TMB-ELISA (Thermo Scientific, #34028) was added and the reaction was stopped 30 min later by adding 2 M sulfuric acid. The absorption at 450 nm was measured with a FLUOstar Omega Microplate Reader (BMG Labtech).

#### Mass spectrometry

The sEVs samples were lysed with in ice-cold lysis buffer [Dulbecco’s phosphate-buffered saline (PBS) supplemented with 1% Triton X-100, 150 mM NaCl, 5 mM EDTA]^47^ and loaded into a 10 K molecular weight cutoff filter unit (PALL) and centrifuged at 14, 500 × g for 15 min. Then proteins were reduced with 100 mM dithiothreitol (DTT, Roche) at 56 °C for 30 min and alkylated in 100 mM iodoacetamide (IAA, TCI) in dark for 1 h. The filter membrane was then rewashed 3 times with 50 mM NH_4_HCO_3_, and the proteins were digested with 500 ng trypsin (Promega) overnight at 37 °C. The resulting tryptic peptides were eluted by centrifugation at 14, 500 × g for 15 min. Another 50 μL of 50 mM NH_4_HCO_3_ was added to the filter membrane, and the solution was centrifuged once more at 14, 500 × g for 15 min. Next, the two eluates were pooled for C18 chromatography resin (zip-tip) desalting. The zip-tip was wet with 80% Acetonitrile (ACN) and 0.1% Formic Acid (FA) before equilibration in water with 0.1% FA. The peptide samples were eluted with 80% ACN and 0.1% FA. The elution was concentrated and dried, and then resuspended with 10 μL 0.1% FA.

For data-independent acquisition mass spectrometry (DIA-MS), the peptides were spiked with iRT internal standard peptide (Biognosys, Switzerland) and analyzed using a nanoLC system (Easy-nLC 1200 system, Thermo Scientific) coupled to a timsTOF SCP (Bruker). Each sample was loaded onto an Acclaim PepMap trap column (75 μm × 2 cm, 3 μm, C18, 100 Å, Thermo Scientific) and then separated on homemade 100 μm × 150 mm C18 column with 1.9 μm particles (Dr. Maisch HPLC GmbH). The flow rate was set at 300 nL/min, and peptides were eluted with the following gradient: 3 to 25% solvent B (0.1% FA in 80% ACN) for 42 min, 25 to 50% solvent B for 4 min, 50 to 100% solvent B for 4 min, then maintained at 100% solvent B for 10 min. The buffer A was made of 0.1% FA. The TimsTOF SCP was operated in dia-PASEF mode with 25 Da window. The dia - PASEF method consisted of one MS1 scan followed by eight dia - PASEF scans, covering a m/z range from 400 to 1,000 and IM of 0.64–1.37 V s. cm^2^. The TIMS accumulation and ramp times were set to 166 ms. Precursors with a charge state of 1+ were excluded. The collision energy was ramped linearly over the ion mobility range, with 20 eV applied at 0.6 V s/cm^2^ to 59 eV at 1.6 V s/cm^2^. The mass spectrometer was operated in high sensitivity mode.

For data-dependent acquisition mass spectrometry (DDA-MS), the peptides were analyzed using a nanoLC system (M class, Waters) coupled to an Orbitrap Exploris 480 Mass Spectrometer (Thermo Fisher Scientific). Each sample was loaded onto an Acclaim PepMap trap column (75 μm × 2 cm, 3 μm, C18, 100 Å, Thermo Scientific) and then separated on a nanoEase BEH C18 column (150 μm × 100 mm, 1.7 μm, C18, 130 Å, Waters). The flow rate was set at 600 nL/min, and the peptides were eluted with the following gradient: 1 to 5% solvent B (0.1% FA in 100% ACN) for 1 min, 5 to 10% solvent B for 2 min, 10 to 30% solvent B for 43 min, 30 to 95% solvent B for 2 min, then maintained at 95% solvent B for 4 min, decreased to 1% solvent B for 1 min, and maintained at 1% solvent B for 7 min. MS1 spectra were collected in the range of 350 to 1,200 m/z at 120,000 resolution to hit an AGC target of 1 × 10^6^. The max injection time of full scan was 30 ms. The top 20 precursor ions with charge states of 2+ to 6+ that exceeded 5 × 10^4^ were selected for fragmentation with normalized collision energies of 30. MS2 spectra were collected in the range of more than 110 m/z at 30,000 resolutions to set an AGC target of 1 × 10^5^. The max injection time was 35 ms. The dynamic exclusion time was set to 15 s.

For DIA data, the files were processed with Spectronaut 18 against the removed pig protein Uniprot database (335,599 entries). Trypsin was selected as the specific enzyme with a maximum of two missed cleavages. Oxidation (M) and acetyl (protein N-term) was set as variable modifications and carbamidomethylation (C) was set as a fixed modification. The label-free quantitation based on extracted ion chromatograms was performed using the direct-DIA module. Proteins were quantified at the MS2 level. The quantitative data were normalized cross all runs. The other parameters were set as default.

For DDA data, the raw files were processed via PEAKS DB database search algorithm of PEAKS online 11 software. The same pig protein Uniprot database (335,599 entries) were used. The mass tolerance was 10 ppm for peptides and 0.02 Da for fragment ions. A maximum of three missed cleavages were allowed for trypsin digestion. Carbamidomethylation (C) was defined as fixed modifications, and oxidation (M) were set as variable modifications. The *de novo* parameters used the same settings of database search parameters. The false discovery rate (FDR) of PSM level was 1%.^48^

By comparing multiple databases, we analyzed the differences in the number of biomarkers contained in sEVs isolated by different purification methods.^12^^,^^36^^,^^37^

### Quantification and statistical analysis

All data, collected from a minimum of 3 biological repeats with each experiment performed with at least three replicates, were expressed as means ± SEM. ELISA, protein concentration, particles/protein ratio were analyzed by one-way ANOVA followed by Bonferroni’s post-hoc analysis for multiple comparisons. The significance threshold was set at *p* ≤ 0.05, ∗*p* ≤ 0.05, ∗∗*p* ≤ 0.01 and ∗∗∗*p* ≤ 0.001. Statistical analysis was performed using GraphPad Prism (GraphPad Software Inc.) or Origin (OriginLab).

## Supplemental information

Document S1. Figures S1–S7

Table S1. Protein abundance of pretreated and no-treated samples detected by DIA-MS

Table S2. Proteins of plasma EVs prepared by different methods detected by MS

Table S3. EV proteins detected by DIA-MS

Table S4. ExoCarta top 100 sEV proteins detected by DIA-MS

Table S5. Abundance of EV markers detected by DIA-MS

## References

[1] Théry C., Witwer K.W., Aikawa E., Alcaraz M.J., Anderson J.D., Andriantsitohaina R., Antoniou A., Arab T., Archer F., Atkin-Smith G.K. (2018). Minimal information for studies of extracellular vesicles 2018 (MISEV2018): a position statement of the International Society for Extracellular Vesicles and update of the MISEV2014 guidelines. J. Extracell. Vesicles.

[2] Colombo M., Raposo G., Théry C. (2014). Biogenesis, secretion, and intercellular interactions of exosomes and other extracellular vesicles. Annu. Rev. Cell Dev. Biol..

[3] Raposo G., Stoorvogel W. (2013). Extracellular vesicles: exosomes, microvesicles, and friends. J. Cell Biol..

[4] Cocucci E., Meldolesi J. (2015). Ectosomes and exosomes: shedding the confusion between extracellular vesicles. Trends Cell Biol..

[5] Yuana Y., Sturk A., Nieuwland R. (2013). Extracellular vesicles in physiological and pathological conditions. Blood Rev..

[6] Irmer B., Chandrabalan S., Maas L., Bleckmann A., Menck K. (2023). Extracellular Vesicles in Liquid Biopsies as Biomarkers for Solid Tumors. Cancers (Basel).

[7] Zhang Y., Wu W., Pan X., Wang Y., Wu C., Lu L., Yu X.-Y., Li Y. (2022). Extracellular vesicles as novel therapeutic targets and diagnosis markers. Extracell. Vesicle.

[8] Padala S.P., Newhouse P.A. (2023). Blood-based biomarkers in Alzheimer's disease: a mini-review. Metab. Brain Dis..

[9] Ramos-Zaldívar H.M., Polakovicova I., Salas-Huenuleo E., Corvalán A.H., Kogan M.J., Yefi C.P., Andia M.E. (2022). Extracellular vesicles through the blood-brain barrier: a review. Fluids Barriers CNS.

[10] Gómez-Río M., Caballero M.M., Górriz Sáez J.M., Mínguez-Castellanos A. (2016). Diagnosis of Neurodegenerative Diseases: The Clinical Approach. Curr. Alzheimer Res..

[11] Li P., Kaslan M., Lee S.H., Yao J., Gao Z. (2017). Progress in Exosome Isolation Techniques. Theranostics.

[12] Welsh J.A., Goberdhan D.C.I., O'Driscoll L., Buzas E.I., Blenkiron C., Bussolati B., Cai H., Di Vizio D., Driedonks T.A.P., Erdbrügger U. (2024). Minimal information for studies of extracellular vesicles (MISEV2023): From basic to advanced approaches. J. Extracell. Vesicles.

[13] Linares R., Tan S., Gounou C., Arraud N., Brisson A.R. (2015). High-speed centrifugation induces aggregation of extracellular vesicles. J. Extracell. Vesicles.

[14] Boriachek K., Islam M.N., Möller A., Salomon C., Nguyen N.T., Hossain M.S.A., Yamauchi Y., Shiddiky M.J.A. (2018). Biological Functions and Current Advances in Isolation and Detection Strategies for Exosome Nanovesicles. Small.

[15] Welton J.L., Webber J.P., Botos L.A., Jones M., Clayton A. (2015). Ready-made chromatography columns for extracellular vesicle isolation from plasma. J. Extracell. Vesicles.

[16] Kalra H., Adda C.G., Liem M., Ang C.S., Mechler A., Simpson R.J., Hulett M.D., Mathivanan S. (2013). Comparative proteomics evaluation of plasma exosome isolation techniques and assessment of the stability of exosomes in normal human blood plasma. Proteomics.

[17] Tóth E.Á., Turiák L., Visnovitz T., Cserép C., Mázló A., Sódar B.W., Försönits A.I., Petővári G., Sebestyén A., Komlósi Z. (2021). Formation of a protein corona on the surface of extracellular vesicles in blood plasma. J. Extracell. Vesicles.

[18] Yuana Y., Levels J., Grootemaat A., Sturk A., Nieuwland R. (2014). Co-isolation of extracellular vesicles and high-density lipoproteins using density gradient ultracentrifugation. J. Extracell. Vesicles.

[19] Huff T., Boyd B., Jialal I. (2023). StatPearls [Internet].

[20] Mathieu M., Martin-Jaular L., Lavieu G., Théry C. (2019). Specificities of secretion and uptake of exosomes and other extracellular vesicles for cell-to-cell communication. Nat. Cell Biol..

[21] Karimi N., Cvjetkovic A., Jang S.C., Crescitelli R., Hosseinpour Feizi M.A., Nieuwland R., Lötvall J., Lässer C. (2018). Detailed analysis of the plasma extracellular vesicle proteome after separation from lipoproteins. Cell. Mol. Life Sci..

[22] Reymond S., Gruaz L., Sanchez J.-C. (2023). Depletion of abundant plasma proteins for extracellular vesicle proteome characterization: benefits and pitfalls. Anal. Bioanal. Chem..

[23] Wagner J., Riwanto M., Besler C., Knau A., Fichtlscherer S., Röxe T., Zeiher A.M., Landmesser U., Dimmeler S. (2013). Characterization of levels and cellular transfer of circulating lipoprotein-bound microRNAs. Arterioscler. Thromb. Vasc. Biol..

[24] O'Brien J., Hayder H., Zayed Y., Peng C. (2018). Overview of MicroRNA Biogenesis, Mechanisms of Actions, and Circulation. Front. Endocrinol..

[25] Bellomo G., Paciotti S., Concha-Marambio L., Rizzo D., Wojdaƚa A.L., Chiasserini D., Gatticchi L., Cerofolini L., Giuntini S., De Luca C.M.G. (2023). Cerebrospinal fluid lipoproteins inhibit alpha-synuclein aggregation by interacting with oligomeric species in seed amplification assays. Mol. Neurodegener..

[26] Takov K., Yellon D.M., Davidson S.M. (2019). Comparison of small extracellular vesicles isolated from plasma by ultracentrifugation or size-exclusion chromatography: yield, purity and functional potential. J. Extracell. Vesicles.

[27] Nieuwland R., Siljander P.R.M. (2024). A beginner's guide to study extracellular vesicles in human blood plasma and serum. J. Extracell. Vesicles.

[28] Onódi Z., Pelyhe C., Terézia Nagy C., Brenner G.B., Almási L., Kittel Á., Manček-Keber M., Ferdinandy P., Buzás E.I., Giricz Z. (2018). Isolation of High-Purity Extracellular Vesicles by the Combination of Iodixanol Density Gradient Ultracentrifugation and Bind-Elute Chromatography From Blood Plasma. Front. Physiol..

[29] Mørk M., Handberg A., Pedersen S., Jørgensen M.M., Bæk R., Nielsen M.K., Kristensen S.R. (2017). Prospects and limitations of antibody-mediated clearing of lipoproteins from blood plasma prior to nanoparticle tracking analysis of extracellular vesicles. J. Extracell. Vesicles.

[30] Rider M.A., Hurwitz S.N., Meckes D.G. (2016). ExtraPEG: A Polyethylene Glycol-Based Method for Enrichment of Extracellular Vesicles. Sci. Rep..

[31] Witwer K.W., Buzás E.I., Bemis L.T., Bora A., Lässer C., Lötvall J., Nolte-’t Hoen E.N., Piper M.G., Sivaraman S., Skog J. (2013). Standardization of sample collection, isolation and analysis methods in extracellular vesicle research. J. Extracell. Vesicles.

[32] Visan K.S., Lobb R.J., Ham S., Lima L.G., Palma C., Edna C.P.Z., Wu L.Y., Gowda H., Datta K.K., Hartel G. (2022). Comparative analysis of tangential flow filtration and ultracentrifugation, both combined with subsequent size exclusion chromatography, for the isolation of small extracellular vesicles. J. Extracell. Vesicles.

[33] Guo J., Wu C., Lin X., Zhou J., Zhang J., Zheng W., Wang T., Cui Y. (2021). Establishment of a simplified dichotomic size-exclusion chromatography for isolating extracellular vesicles toward clinical applications. J. Extracell. Vesicles.

[34] Freitas D., Balmaña M., Poças J., Campos D., Osório H., Konstantinidi A., Vakhrushev S.Y., Magalhães A., Reis C.A. (2019). Different isolation approaches lead to diverse glycosylated extracellular vesicle populations. J. Extracell. Vesicles.

[35] Wang X., Wang F., Arterburn L., Wollmann R., Ma J. (2006). The interaction between cytoplasmic prion protein and the hydrophobic lipid core of membrane correlates with neurotoxicity. J. Biol. Chem..

[36] Keerthikumar S., Chisanga D., Ariyaratne D., Al Saffar H., Anand S., Zhao K., Samuel M., Pathan M., Jois M., Chilamkurti N. (2016). ExoCarta: A Web-Based Compendium of Exosomal Cargo. J. Mol. Biol..

[37] Hoshino A., Kim H.S., Bojmar L., Gyan K.E., Cioffi M., Hernandez J., Zambirinis C.P., Rodrigues G., Molina H., Heissel S. (2020). Extracellular Vesicle and Particle Biomarkers Define Multiple Human Cancers. Cell.

[38] Thery C., Amigorena S., Raposo G., Clayton A. (2006). Isolation and characterization of exosomes from cell culture supernatants and biological fluids. Curr Protoc Cell Biol Chapter.

[39] Wu Q., Cortez L., Kamali-Jamil R., Sim V., Wille H., Kar S. (2021). Implications of exosomes derived from cholesterol-accumulated astrocytes in Alzheimer's disease pathology. Dis. Model. Mech..

[40] Simonsen J.B. (2017). What Are We Looking At? Extracellular Vesicles, Lipoproteins, or Both?. Circ. Res..

[41] Blaser M.C., Buffolo F., Halu A., Turner M.E., Schlotter F., Higashi H., Pantano L., Clift C.L., Saddic L.A., Atkins S.K. (2023). Multiomics of Tissue Extracellular Vesicles Identifies Unique Modulators of Atherosclerosis and Calcific Aortic Valve Stenosis. Circulation.

[42] Yan H., Li Y., Cheng S., Zeng Y. (2021). Advances in Analytical Technologies for Extracellular Vesicles. Anal. Chem..

[43] Wu Q., Karthivashan G., Nakhaei-Nejad M., Anand B.G., Giuliani F., Kar S. (2022). Native PLGA nanoparticles regulate APP metabolism and protect neurons against beta-amyloid toxicity: Potential significance in Alzheimer's disease pathology. Int. J. Biol. Macromol..

[44] Barranco I., Alvarez-Barrientos A., Parra A., Martínez-Díaz P., Lucas X., Roca J. (2024). Immunophenotype profile by flow cytometry reveals different subtypes of extracellular vesicles in porcine seminal plasma. Cell Commun. Signal..

[45] Kibria G., Ramos E.K., Lee K.E., Bedoyan S., Huang S., Samaeekia R., Athman J.J., Harding C.V., Lötvall J., Harris L. (2016). A rapid, automated surface protein profiling of single circulating exosomes in human blood. Sci. Rep..

[46] Abskharon R., Wang F., Wohlkonig A., Ruan J., Soror S., Giachin G., Pardon E., Zou W., Legname G., Ma J. (2019). Structural evidence for the critical role of the prion protein hydrophobic region in forming an infectious prion. PLoS Pathog..

[47] Bargar C., Wang W., Gunzler S.A., LeFevre A., Wang Z., Lerner A.J., Singh N., Tatsuoka C., Appleby B., Zhu X. (2021). Streamlined alpha-synuclein RT-QuIC assay for various biospecimens in Parkinson's disease and dementia with Lewy bodies. Acta Neuropathol. Commun..

[48] Wiśniewski J.R., Zougman A., Nagaraj N., Mann M. (2009). Universal sample preparation method for proteome analysis. Nat. Methods.

