# Supplementary material for: Optimizing plasma-derived small extracellular vesicle purification to enhance downstream applications
Source: iScience. 2026 Aug 22;29(9):117286. doi: 10.1016/j.isci.2026.117286 (PMC13524700; doi:10.1016/j.isci.2026.117286)
Supplement: Supplementary file 1 [file mmc1.pdf]

iScience, Volume *29*

Supplemental information

Optimizing plasma-derived small extracellular vesicle purification to enhance downstream applications

Fanxiu Ma, Meng Yuan, Yue Zhao, Xiumei Jin, Chengsi Wu, Xin Fang, Zhaodi Jiang, Shuying Liu, Wen-Quan Zou, Zhen Li, Qi Wu, and Jiyan Ma

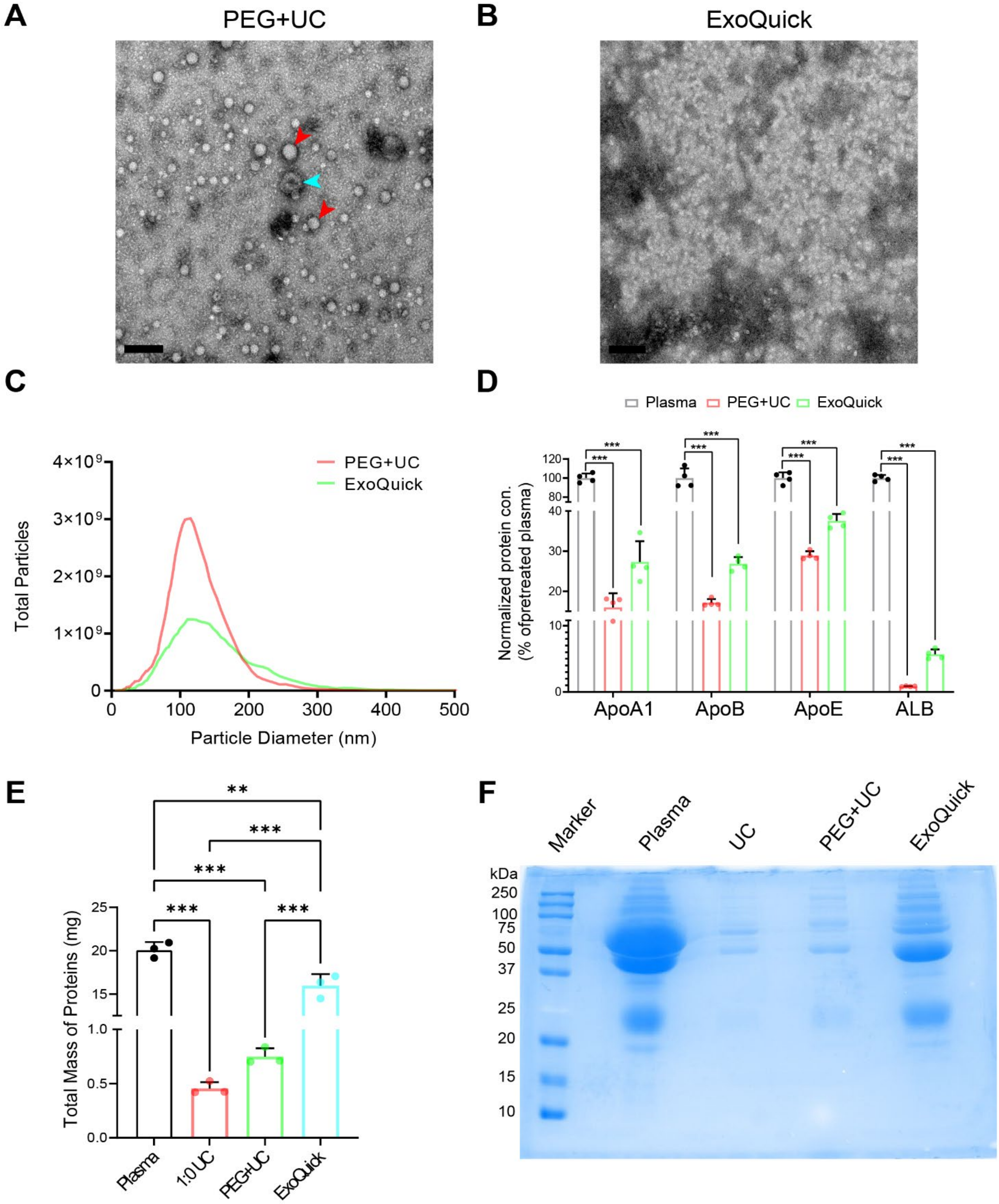

Supplementary figure 1. Precipitation based methods are not well suited for plasma sEVs isolation. Small EVs were isolated by polyethylene glycol (PEG) based precipitation and washed by ultracentrifugation (PEG+UC) or using ExoQuick exosome precipitation kit as indicated. The morphology of sEVs isolated by PEG+UC (A) or ExoQuick (B) were detected by TEM. Lipoproteins were indicated by red arrow heads; cyan arrow head indicated a sEV. (C) NTA of total particles isolated by indicated methods. Average size of particles: PEG+UC – 112.2±43 nm, ExoQuick – 125.3±57.9 nm. (D) Histogram indicated the percentage of ApoA1, ApoB, ApoE and ALB remained after indicated treatments. (E and F) Proteins in particles isolated by PEG+UC or ExoQuick were determined by BCA protein assay (E) and Coomassie blue staining (F). Statistical significance was assessed using one-way ANOVA. Data are represented as mean ± SEM, ** $p \leq 0.01$ and *** $p \leq 0.001$. Scale bar: 200 nm.

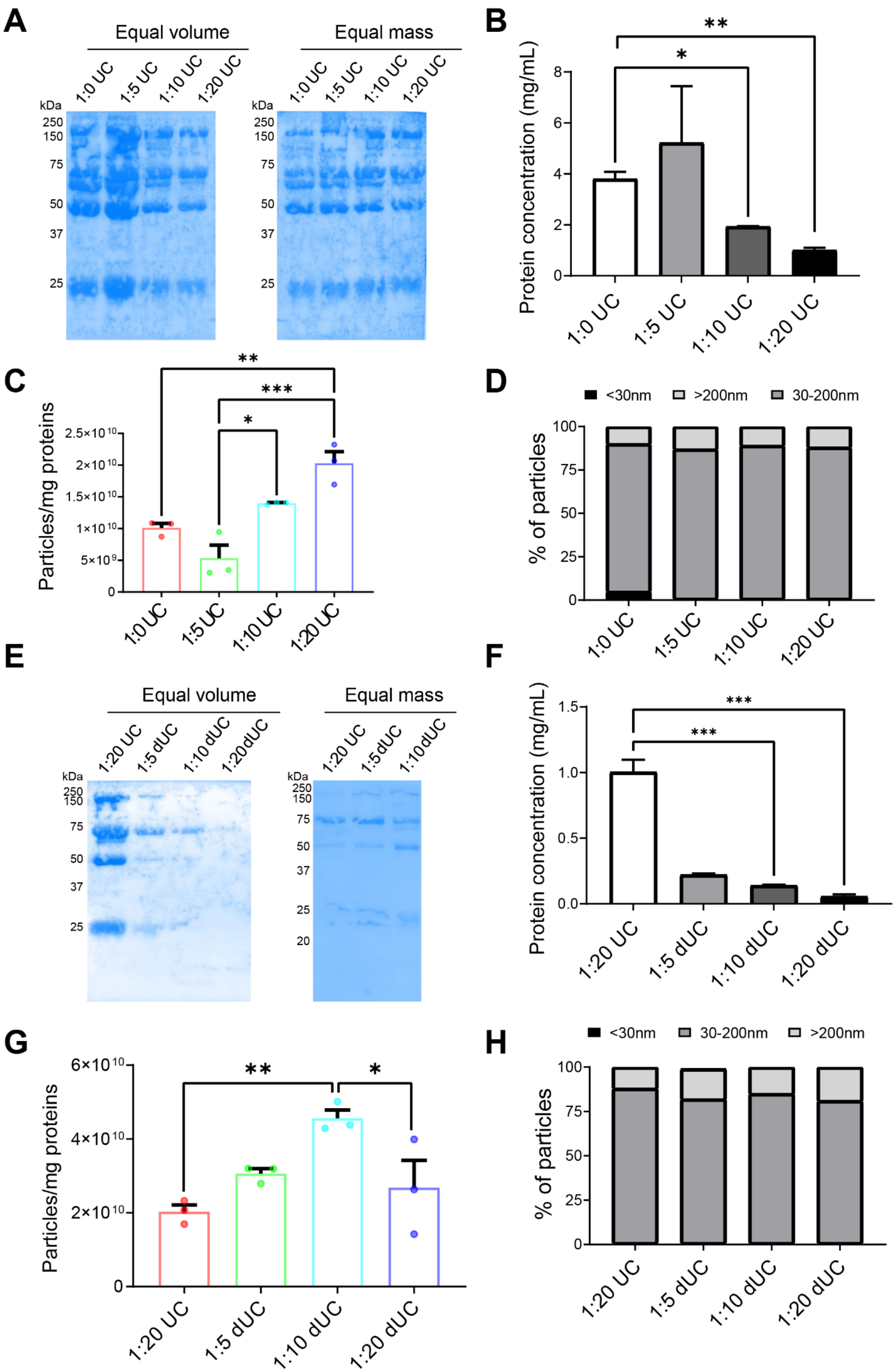

A
Equal volume
Equal mass
1:0 UC
1:5 UC
1:10 UC
1:20 UC
kDa
250
150
75
50
37
25
B
Protein concentration (mg/mL)
**
*
C
Particles/mg proteins
2.5×10^{10}
2×10^{10}
1.5×10^{10}
1×10^{10}
5×10^{9}
***
D
<30nm
>200nm
30-200nm
% of particles
E
1:20 UC
1:5 dUC
1:10 dUC
1:20dUC
20
F
1:20 dUC
G
6×10^{10}
4×10^{10}
2×10^{10}
H

Supplementary figure 2. Protein levels in sEVs isolated by UC or dUC. Proteins in sEVs isolated by UC (A-D) and dUC (E-H) were determined by Coomassie blue staining (A and E) and protein concentration were measured by BCA protein assay (B and F). (C and G) Histogram indicated the particles/proteins ratio of sEVs. (D and H) Histogram indicated the percentage of particle size ranges. Statistical significance was assessed using one-way ANOVA. Data are represented as mean ± SEM, * $p \leq 0.05$ and ** $p \leq 0.01$.

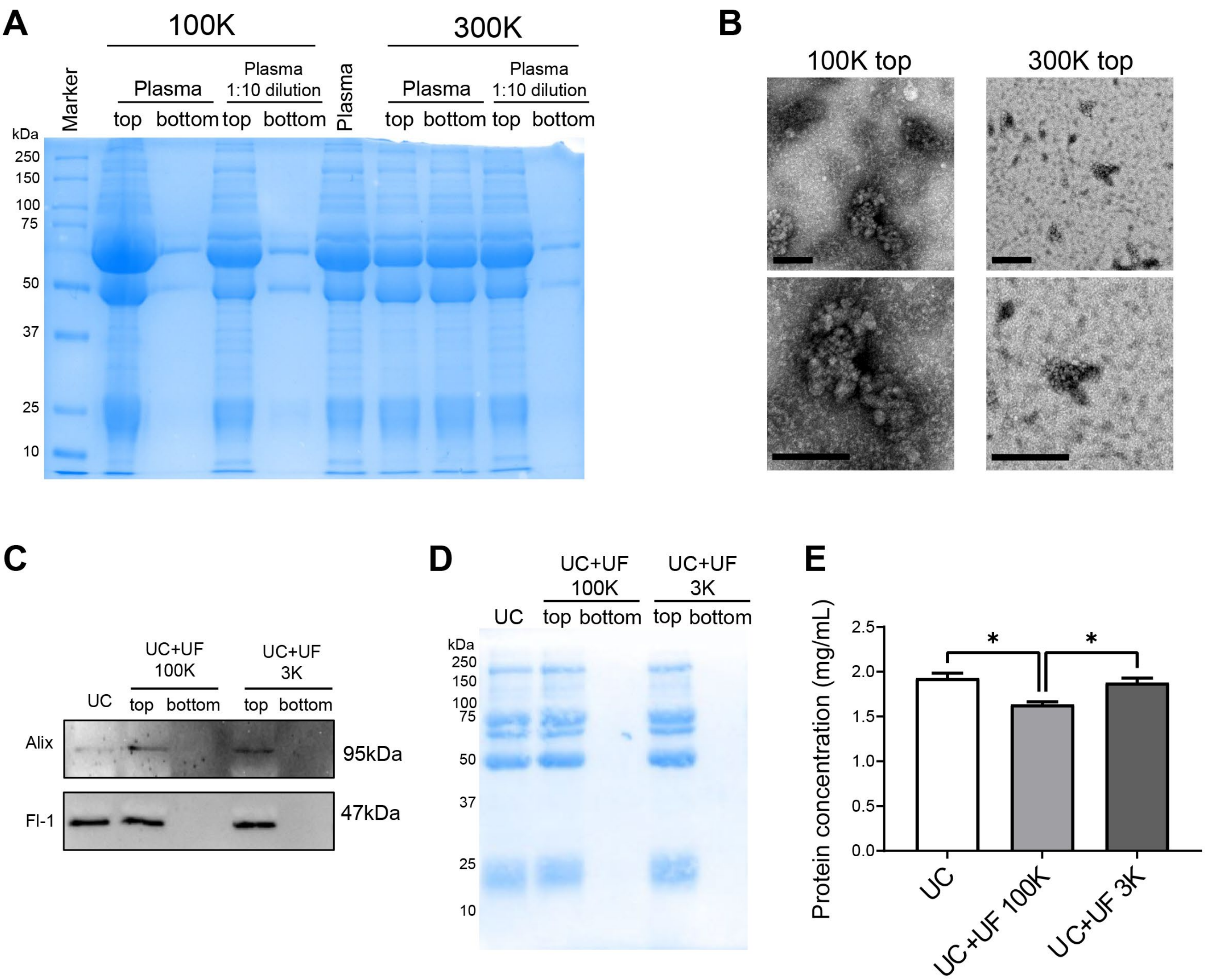

Supplementary figure 3. Ultrafiltration efficiently concentrates sEVs but is not suitable for directly isolating sEVs from plasma. (A and B) Undiluted or 1:10 diluted pretreated pig plasma were loaded in 100K or 300K centrifugal filter unit. (A) The top fractions and bottom fractions were loaded in SDS-PAGE and stained by Coomassie blue. (B) The morphology of sEVs in the top fractions were detected by TEM. (C-E) 1:20 UC pellets were resuspended with 500μL of PBS and concentrated by 100K or 3K centrifugal filter unit. Scale bar: 200 nm. (C) Small EVs were characterized by immunoblotting of indicated sEVs protein markers. (D and E) 1:20 UC pellet were resuspended in 120 μL (UC) or resuspended in 500 μL PBS and concentrated to 120 μL using 3 kDa (3K) or 100 kDa (100K) filters as indicated. Proteins in sEVs isolated by indicated methods were determined by Coomassie blue staining (D) and protein concentration were measured by BCA protein assay (E). Statistical significance was assessed using one-way ANOVA. Data are represented as mean ± SEM, * $p \leq 0.05$.

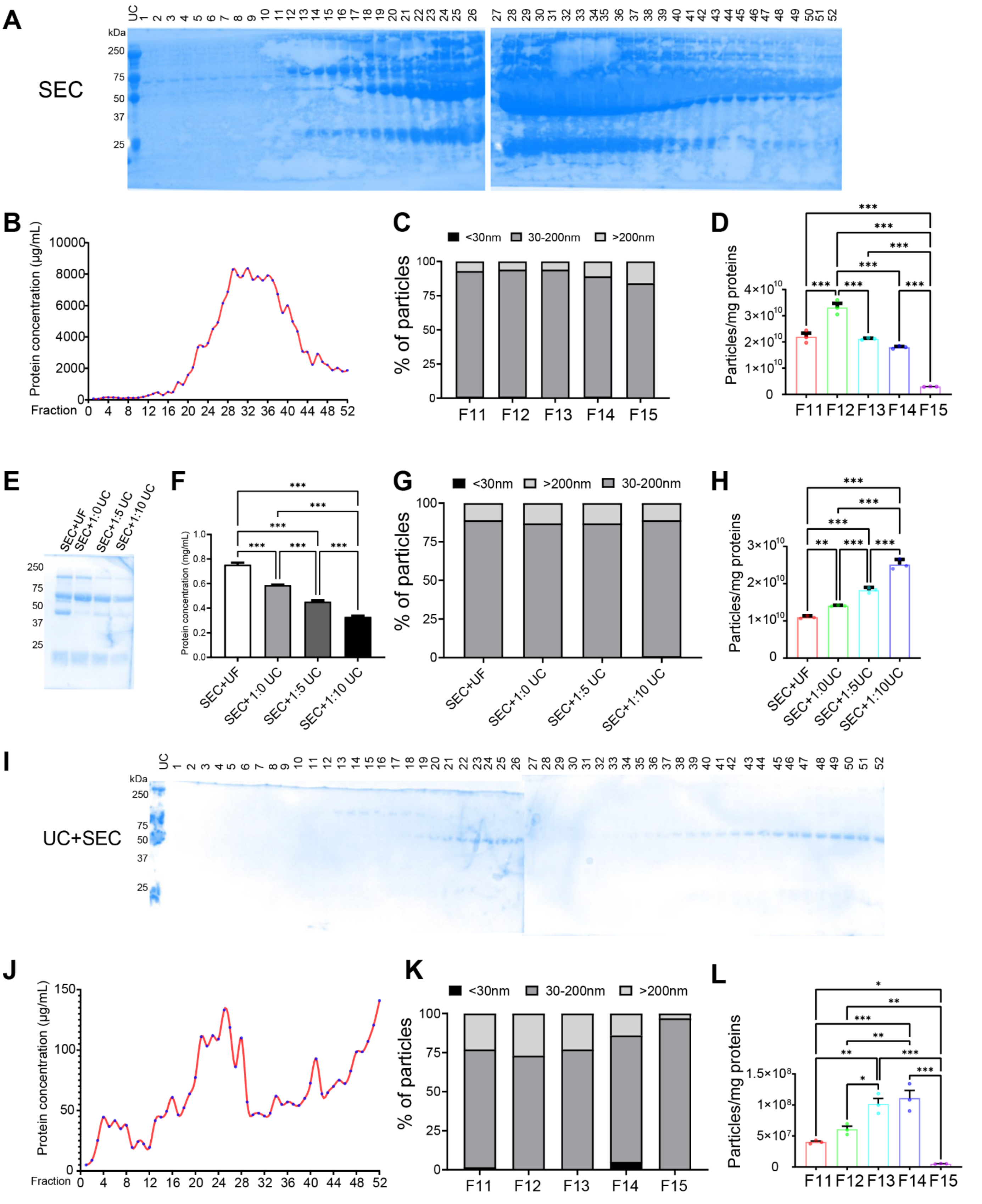

Supplementary figure 4. Protein levels in sEVs isolated by SEC. Proteins in 52 SEC fractions (A and B), concentrated SEC 11-15 fractions (E and F), and 52 UC+SEC fractions (I and J) were determined by Coomassie blue staining (A, E and I) and BCA protein assay (B, F and J). (C, G and K) Histograms indicated the percentage of particles of various sizes. (D, H and L) Histogram indicated the particle/protein ratio of sEVs. Statistical significance was assessed using one-way ANOVA. Data are represented as mean ± SEM, * $p \leq 0.05$, ** $p \leq 0.01$ and *** $p \leq 0.001$.

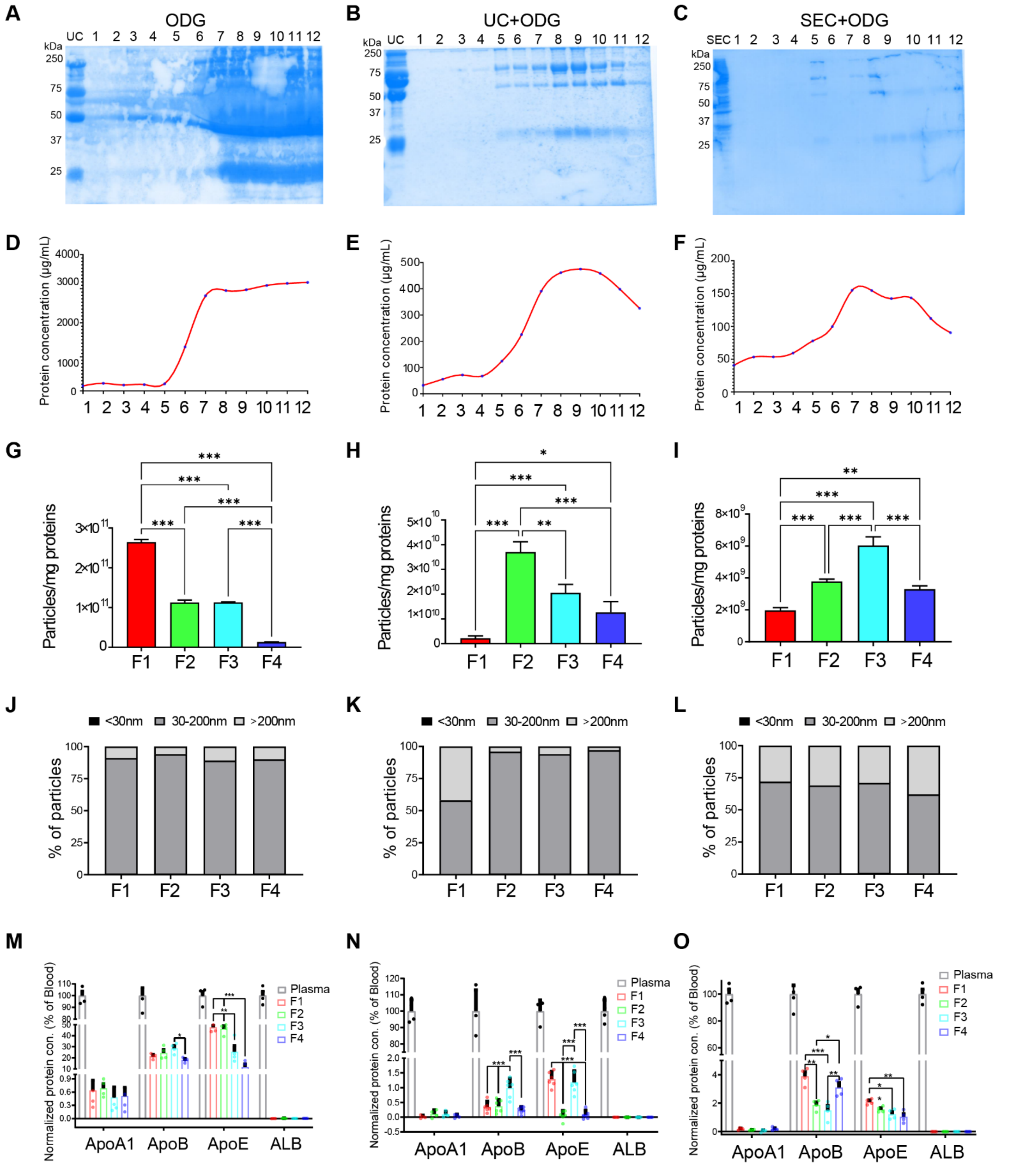

Supplementary figure 5. Protein levels in sEVs isolated by ODG. (A-F) Proteins in ODG sEVs (A and D), UC+ODG sEVs (B and E), and SEC+ODG sEVs (C and F) were determined by Coomassie blue staining (A-C) and BCA protein assay (D-F). (G-I) Histogram indicated the particle/protein ratio of sEVs. (J-L) Histograms indicated the percentage of particles of various sizes es in ODG, UC+ODG, SEC+ODG sEVs. (M-O) Histogram indicated the percentage of ApoA1, ApoB, ApoE and ALB remained after indicated treatments. Statistical significance was assessed using one-way ANOVA. Data are represented as mean ± SEM, * $p \leq 0.05$, ** $p \leq 0.01$ and *** $p \leq 0.001$.

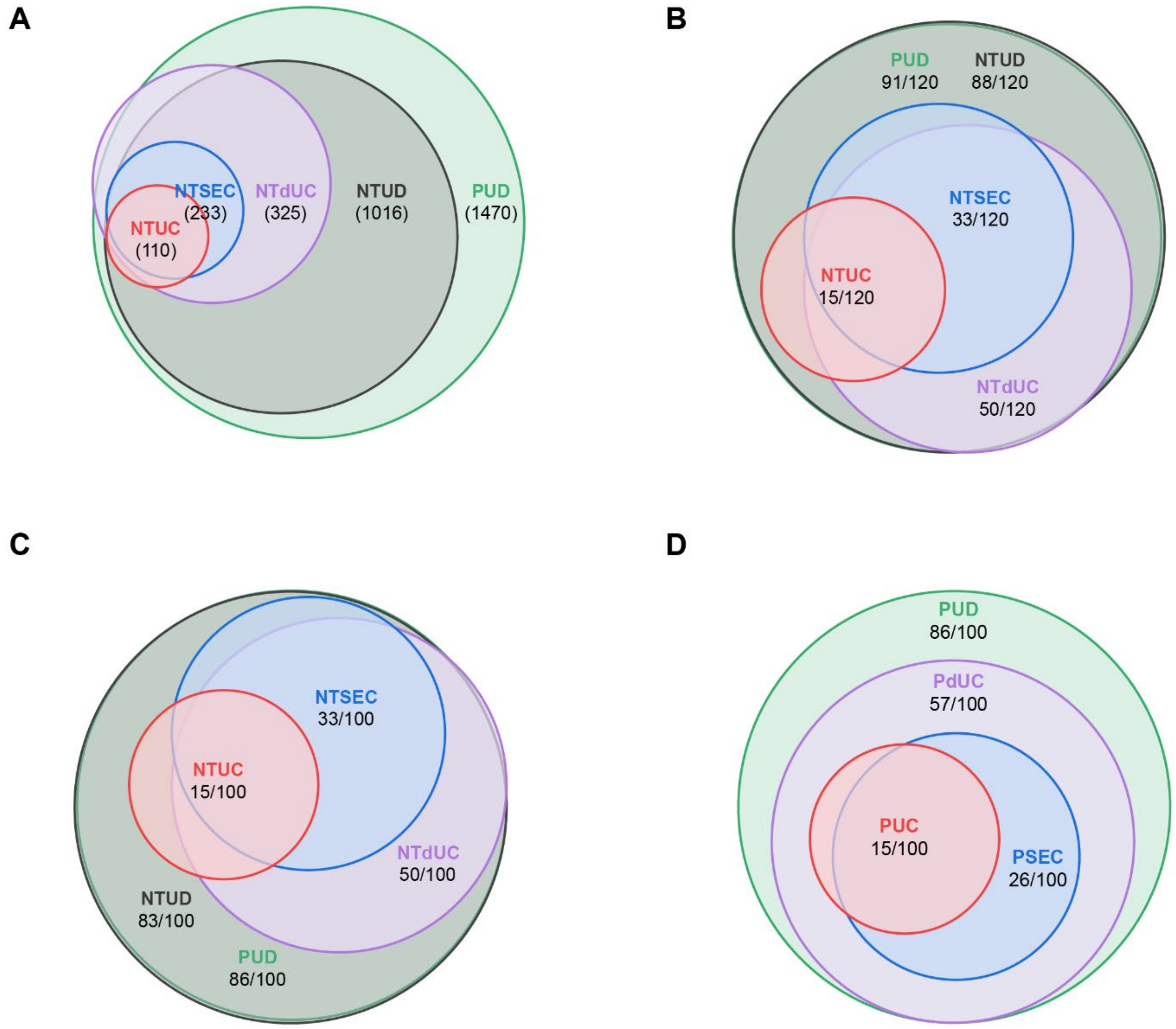

Supplementary figure 6. EVs isolated by PUD and NTUD recovered a greater number of proteins and EV markers in comparison with EVs isolated by other methods. EV proteins were detected by mass spectrometry (DIA-MS). Venn diagram indicated the number of proteins identified (A), the number of commonly detected sEV proteins identified (B), the number of ExoCarta top 100 sEV proteins identified (C and D) in sEVs isolated from pre-treated plasma with 1:10 UC, 1:10 dUC, SEC, or UC+ODG. NT, non-treated; P, pre-treated.

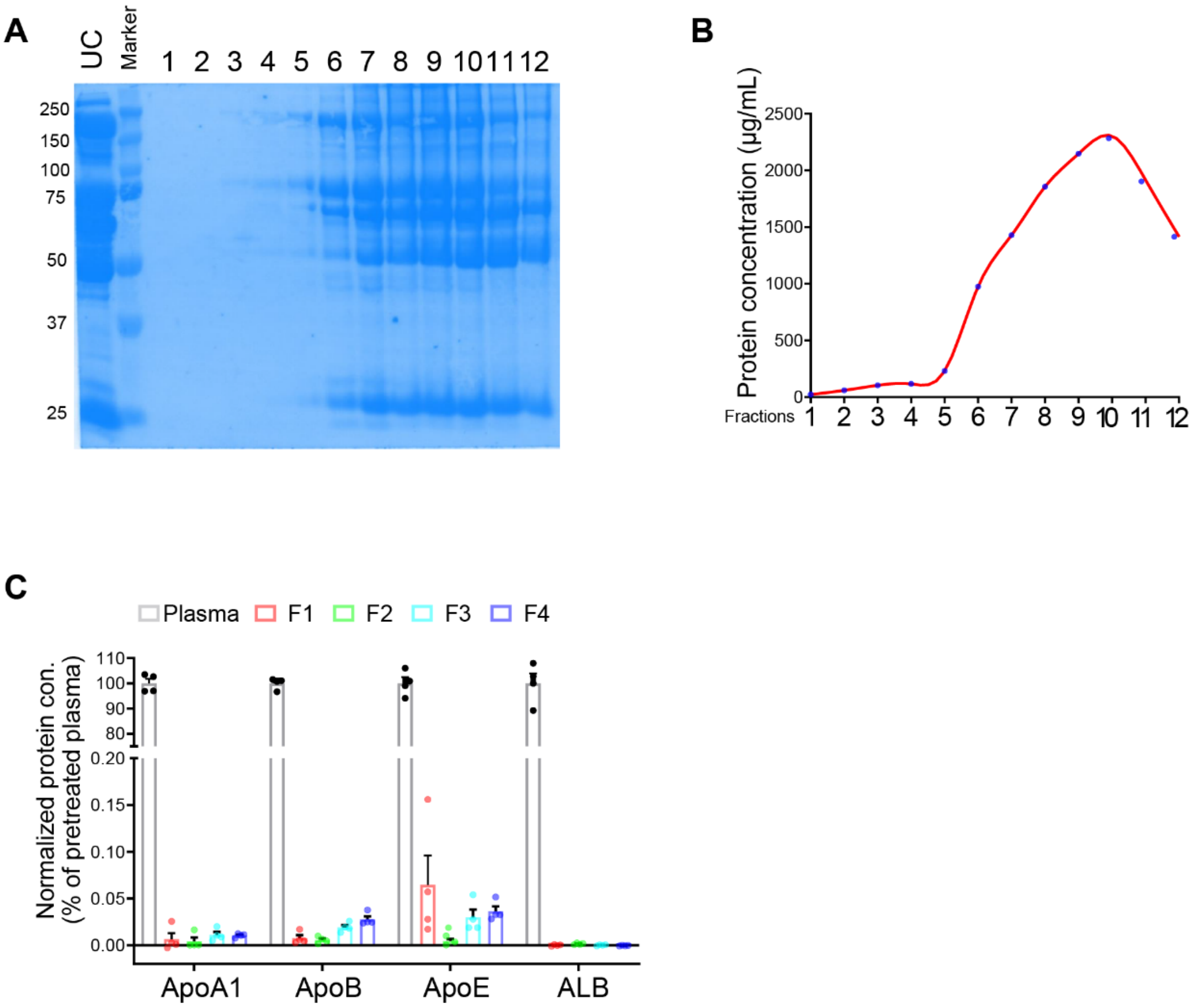

Supplementary figure 7. Protein levels in human plasma sEVs isolated by PUD. Proteins in human PUD fractions1-12 were determined by Coomassie blue staining (A) and protein concentration were measured by BCA protein assay (B). (C) Histogram indicated the percentage of ApoA1, ApoB, ApoE and ALB remained in the PUD fractions F1-F4.
